# Effect of RYGB Limb Lengths on HbA1c in Patients with Obesity and Type 2 Diabetes

**DOI:** 10.1007/s11695-026-08690-6

**Published:** 2026-04-21

**Authors:** Lena Seidemann, Roland Morgenroth, Yusef Moulla, Undine Gabriele Lange, Orestis Lyros, Matthias Blüher, David Petroff, Christiane Prettin, Norbert Köhler, Arne Dietrich

**Affiliations:** 1 Department of Visceral, Transplant, Thoracic and Vascular Surgery, Leipzig University Hospital, Leipzig, Germany; 2 Integrated Research and Treatment Center (IFB) Adiposity Diseases, Leipzig University Hospital, Leipzig, Germany; 3 Department of General and Visceral Surgery, Medical Campus Chemnitz of TU Dresden, Chemnitz, Germany; 4 Fourth Department of Surgery, Attikon University Hospital, National and Kapodistrian University of Athens, Haidari, Greece; 5 Department of Endocrinology, Nephrology, Rheumatology, Leipzig University Hospital, Leipzig, Germany; 6 Helmholtz Institute for Metabolic, Obesity and Vascular Research (HI-MAG) of the Helmholtz Zentrum München at the University of Leipzig and University Hospital Leipzig, Leipzig, Germany; 7 Clinical Trial Centre, University of Leipzig, Leipzig, Germany

**Keywords:** Roux-en-Y gastric bypass, Small bowel limb lengths, Long biliopancreatic limb, Type 2 diabetes, Metabolic surgery

## Abstract

**Introduction:**

Roux-en-Y gastric bypass (RYGB) is one of the most commonly performed procedures in metabolic surgery, known for its beneficial effects on type 2 diabetes (T2D). However, the optimal choice of small bowel limb lengths remains a matter of debate. Recent studies suggest that a longer biliopancreatic limb (BPL) could improve T2D more effectively than a longer alimentary limb (AL). The aim of this randomized controlled trial was to compare the effects of a long BPL-RYGB to a long AL-RYGB on T2D in adults with body mass index (BMI) between 27 and 60 kg/m^2^.

**Methods:**

131 patients were randomized 1:1 to long AL-RYGB (150 cm AL/50 cm BPL) or long BPL-RYGB (50 cm AL/150 cm BPL). In patients with BMI > 50 kg/m^2^, limb lengths were slightly longer. The primary outcome was glycated hemoglobin (HbA1c) after 12 months. Secondary outcomes were T2D remission rate, weight loss, anthropometric measures and blood parameters reflecting glycemic control, dyslipidemia and micronutrient supply. Trial registration number: DRKS00007810 (German Clinical Trials Register Freiburg).

**Results:**

Patients in the long BPL-RYGB group showed a -0.33% greater decrease in HbA1c (95% CI [-0.60, -0.06], *p* = 0.018). Further advantages of long BPL-RYGB were found for improvements in fasting insulin, body weight, BMI and waist-to-hip ratio. No statistically significant differences were observed regarding T2D remission rate, fasting glucose, fasting c-peptide, HOMA-IR index and blood values for dyslipidemia and micronutrients.

**Conclusion:**

Patients with obesity and T2D may benefit from a long BPL-RYGB in terms of a greater decrease in HbA1c and weight loss without signals for a greater risk of malnutrition, while diabetes remission rates were comparable between procedures at 12 months.

**Supplementary Information:**

The online version contains supplementary material available at 10.1007/s11695-026-08690-6.

## Key Points

Decrease in HbA1c was greater after long BPL-RYGB than after long AL-RYGB.

Body weight and BMI decreased more after long BPL-RYGB than after long-AL RYGB.

Long BPL-RYGB did not lead to a greater risk of malnutrition than long AL-RYGB.

**Supplementary Information:**

The online version contains supplementary material available at 10.1007/s11695-026-08690-6.

## Introduction

The global rise in obesity is at the root of the worldwide increasing prevalence of type 2 diabetes (T2D), a leading cause of death and disability [1]. Metabolic-bariatric surgery (MBS) effectively improves and reverses T2D in patients with obesity [2]. One of the most common procedures in MBS is Roux-en-Y gastric bypass (RYGB) [3], which exhibits very good T2D remission in combination with a low rate of nutritional and surgical complications [2, 4, 5]. Although RYGB is considered a standard procedure, it is performed in various different manners [6]. The 2019 consensus statement of the International Federation for the Surgery of Obesity and Metabolic Disorders (IFSO) defined lengths of the alimentary limb (AL) and the biliopancreatic limb (BPL) of 50–150 cm with the constraint that their combined length should be 200 cm [7]. A global survey among metabolic-bariatric surgeons reported that 87% apply constant limb lengths; with a 76–100 cm BPL and a 126–150 cm AL being the most commonly used. When surgeons were asked for factors motivating them to alter limb lengths, patient weight and presence of T2D were the most common answers [6]. However, current evidence on the effect of RYGB limb lengths on outcomes is heterogeneous and most of the available studies were designed to assess weight loss and not T2D as primary endpoint [8]. A recent meta-analysis indicates that the application of a longer BPL is associated with higher T2D improvement rates in comparison to a longer AL [9]. 

The MetaSurg (Metabolic Surgery for Type 2 Diabetes within BMI range of 27 to 60 kg/m^2^) study was designed as a single-center, open-label randomized clinical trial (RCT) to compare the efficacy of two different types of RYGB with either a long AL or a long BPL on T2D with glycated hemoglobin (HbA1c) at 12 months as the primary endpoint.

## Methods

### Trial Design, Participants and Randomization

The MetaSurg study was an open-label, prospective, randomized trial conducted between June 13, 2016 and December 13, 2022 at a University Hospital. Patients with obesity and T2D eligible for MBS were screened at the local obesity treatment center. Inclusion criteria were: body mass index (BMI, calculated as weight in kilograms divided by height in meters squared) ≥ 27 or ≤ 60 kg/m^2^, age ≥ 18 years, and confirmed diagnosis of T2D. For BMI < 35 kg/m^2^, additional criteria were: unsatisfactory medical non-insulin treatment (multiple medications, HbA1c > 6.5% or recurrent hypo- or hyperglycemias), or planned/ongoing insulin treatment. For BMI < 40 kg/m^2^, prior failed conservative treatment was obligatory according to the German MBS guidelines [10]. Exclusion criteria were any chronic inflammatory/malignant disease, type 1 diabetes, peptic ulcer, contraindication for general anesthesia, alcohol or substance abuse, untreated thyroid dysfunction, pregnancy, breastfeeding, non-compliance, and participation in another interventional trial. Previous gastric banding was retrospectively defined as an exclusion criterion. This extension of the exclusion criteria was carried out prior to statistical data analysis.

The original trial design aimed to randomize patients into two surgical arms and an arm treated with a standardized conservative diabetes therapy. However, due to insufficient recruitment and high dropout in the non-surgical arm, randomization was limited to the two surgical arms. The data of the surgical groups are reported here. Computer-assisted randomization was carried out in a 1:1 ratio by the Clinical Trial Centre, using Pocock’s minimization algorithm with a stochastic component. Stratification parameters were: BMI (< 50/≥50 kg/m^2^), insulin treatment (yes/no), and sex (male/female). The trial protocol is presented in Supplement 1.

The study was registered at German Clinical Trials Register Freiburg (ID: DRKS00007810) and approved by the University’s ethics committee (153/15-ek). It was conducted in accordance with the Declaration of Helsinki and Good Clinical Practice, and was monitored by the University’s Clinical Trial Centre. All participants provided written informed consent.

### Interventions

**Fig. 1:**
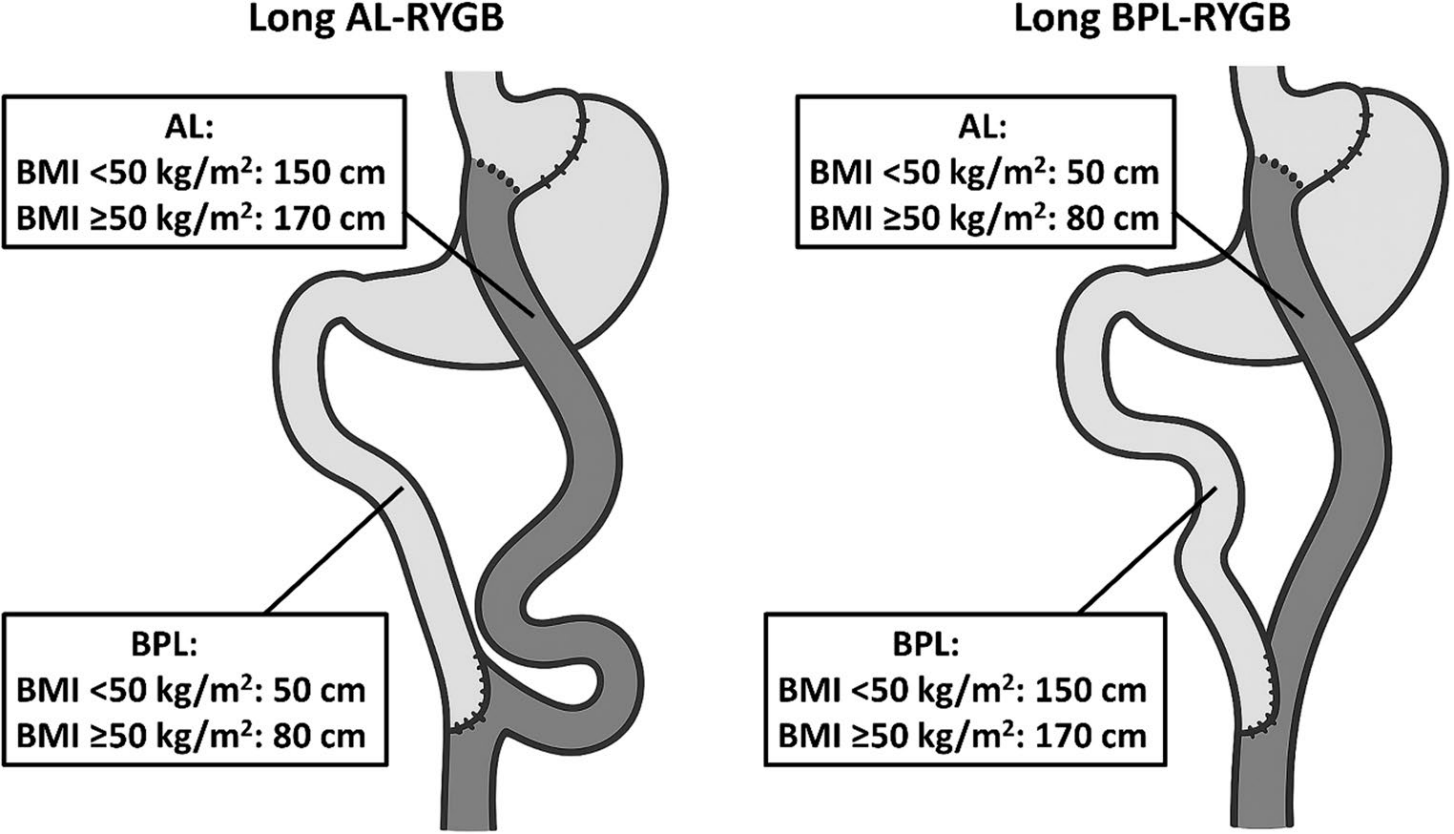
Applied limb lengths in the two surgical groups. Abbreviations: AL, alimentary limb; BPL, biliopancreatic limb; BMI, body mass index; RYGB, Roux-en-Y gastric bypass. Figure created with the assistance of AI-based image generation (ChatGPT, OpenAI)

Laparoscopic RYGB was performed by experienced metabolic-bariatric surgeons using a small gastric pouch (10–20 cc). In the long AL-RYGB group, limb lengths were 150 cm AL/50 cm BPL for BMI ≤ 50 kg/m^2^ and 170 cm AL/80 cm BPL for BMI ≥ 50 kg/m^2^. In the long BPL-RYGB group, limb lengths were 50 cm AL/150 cm BPL for BMI ≤ 50 kg/m^2^ and 80 cm AL/170 cm BPL for BMI ≥ 50 kg/m^2^ (Fig. 1). Accurate measurement of the small bowel was achieved by carefully stretching it between two laparoscopic graspers and holding a third grasper with 5 and 10 cm marks next to it. All patients followed a 14-day hypocaloric, protein-rich diet preoperatively, as previously published by our group and part of standard preoperative treatment at our center [11]. After surgery, patients were instructed to take multivitamins, vitamin B12, vitamin D, calcium, and iron supplements.

#### Outcomes

The primary endpoint was HbA1c at 12 months. T2D remission rate at 12 months, defined as HbA1c <6.5% without anti-diabetic medication, was a secondary endpoint. Further secondary endpoints included: (1) anthropometric measures (body weight, BMI, waist-to-hip-ratio, waist-to-height-ratio), and (2) blood parameters (fasting glucose, insulin, c-peptide, HOMA-IR (homeostatic model assessment for insulin resistance), total cholesterol, HDL (high density lipoprotein), LDL (low density lipoprotein), triglycerides, iron, albumin, zinc, and vitamin D3), all assessed at 6 and 12 months. Adverse events were recorded at each study visit. 

#### Sample Size and Statistical Analysis

Sample size calculations were based on the 12-month data from the STAMPEDE trial and assumed a mean (SD) HbA1c reduction of 2.9 (1.7) percentage points in the pooled surgical group vs 1.4 (1.5) in the conservative group [12]. With 120 patients analyzed in the surgical groups and 20 in the conservative group a power of 95% at a 5% significance level was calculated for the comparison between surgical and conservative groups. The sample size was not based on a comparison within the surgical groups, but a width for the 95% confidence interval (CI) of 1.2 percentage points was estimated for this comparison. 

The primary endpoint analysis was based on the per protocol (PP) population using a linear regression model with baseline HbA1c as a covariate and treatment arm and stratification categories (baseline BMI, insulin treatment, and sex) as factors. The PP population included randomized patients who (1) received one of the two interventions, (2) provided data for the primary endpoint at baseline and 12 months, and (3) had no prior gastric banding. 95% CIs and p-values were computed using a Wald t-distribution approximation. 

Secondary endpoints were also analyzed for the PP population. T2D remission rates between the two arms were compared using Pearson’s Chi-squared test with Yates’ continuity correction. Missing data on remission were treated as no remission. The relative probability (“risk”) of T2D remission was estimated with a generalized linear model with a “log” link function, including stratification variables as covariates. Differences in clinical, anthropometric, and blood parameters were analyzed using linear mixed-effects models with treatment arm, time (categorical), and stratification categories as fixed effects and a random intercept for subject. An interaction was modelled for treatment arm and time. Fasting insulin, c-peptide, Homeostatic Model Assessment for Insulin Resistance (HOMA-IR) and triglycerides were analyzed on a logarithmic scale. 

Statistical analyses of endpoints were performed using *R* (version ≥4.3.1; R Core Team, 2023) with additional software packages (see Supplement 2, Table S1).

## Results

### Trial participants

**Fig. 2:**
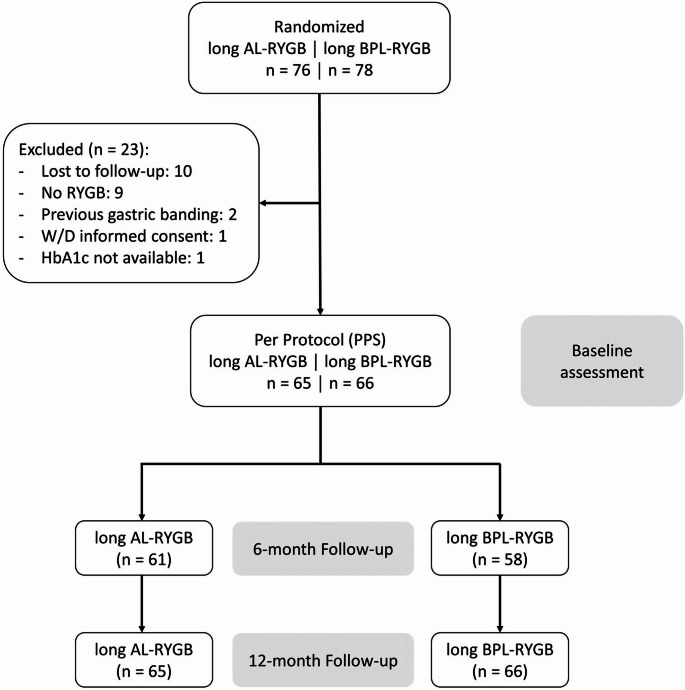
CONSORT flow diagram

Of the 154 patients randomized between June 2016 and December 2021, 23 (11 in the long AL-RYGB group, 12 in the long BPL-RYGB group) either did not receive the assigned treatment or were excluded from the final analyses for the following reasons: one patient in the long BPL-RYGB group withdrew immediately after randomization, 10 patients (5 in each group) were lost to follow-up before the 12-month study visit; in 9 patients (5 in the long AL-RYGB group, 4 in the long BPL-RYGB group) RYGB was not possible for anatomical reasons (formation of a gastrojejunostomy was deemed too risky due to high accumulation of visceral fat) and a sleeve gastrectomy was performed instead; 2 patients from the long BPL-RYGB group were excluded due to prior gastric banding (retrospectively defined as an exclusion criterion); and one patient in the long AL-RYGB group lacked a 12-month HbA1c value (Fig. 2).

After these exclusions, 131 patients were included into the per protocol analysis (Table 1). Among those, 64% were women, mean (±SD) age was 53.8±9.6 years, mean BMI was 46.4±6.2 kg/m^2^, and mean HbA1c 7.5±1.6%. Oral antidiabetic medications were used by 91%, and 56% were treated with insulin.

**Table 1:** Baseline characteristics and postoperative outcomes of patients in the long AL-RYGB and long BPL-RYGB groups

Outcome			long AL-RYGB vs. long BPL-RYGB at follow-up^1^		
	long AL-RYGB (*n* = 65)	long BPL-RYGB (*n* = 66)	Estimate	95% CI	*p*-value
Sex, No. (%)					
Female	42 (65%)	42 (64%)			
Male	23 (35%)	24 (36%)			
Age, mean (SD), y	53.2 (10.0)	54.4 (9.1)			
Height, mean (SD), cm	169.0 (8.7)	170.6 (10.6)			
HbA1c, mean (SD), %					
Baseline	7.8 (1.7)	7.3 (1.4)			
6 months^a^	6.3 (1.0)	5.8 (0.7)			
12 months	6.2 (1.0)	5.7 (0.7)	-0.33	-0.6, -0.06	0.018
Insulin treatment at baseline, No. (%)	39 (60%)	34 (52%)			
Oral antidiabetics at baseline, No. (%)^a^	56 (90%)	60 (91%)			
No anti-diabetic medication at baseline, No. (%)^a^	2 (3.2%)	2 (3.0%)			
T2D remission^2^ rate, % (95% CI)					
12 months	49.2 [37.3, 61.2]^b^	64.1 [51.8, 74.7]^c^	1.29^3^	0.94, 1.79	0.113
Body weight, mean (SD), kg					
Baseline	132.0 (20.8)	136.1 (23.6)			
6 months^d^	100.5 (16.7)	102.1 (19. 5)	-2.5^*^	-5.5, 0.5	0.100
12months^e^	94.1 (16.9)	94.3 (19.5)	-4.1^*^	-7.0, -1.1	0.008
BMI, mean (SD), kg/m^2^					
Baseline	46.1 (5.9)	46.7 (6.4)			
6 months^d^	35.2 (4.9)	35.1 (5.4)	-0.7^*^	-1.7, 0.3	0.171
12 months^e^	32.9 (5.4)	32.3 (5.4)	-1.3^*^	-2.2, -0.3	0.012
BMI, categorical, No. (%)					
< 50 kg/m²	50 (77%)	44 (67%)			
≥ 50 kg/m²	15 (23%)	22 (33%)			
Waist circumference, mean (SD), cm^d^	137.1 (12.9)	139.9 (15.0)			
Hip circumference, mean (SD), cm^d^	139.3 (12.4)	142.5 (15.7)			
Waist-to-hip ratio, mean (SD)					
Baseline^d^	0.99 (0.1)	0.99 (0.1)			
6 months^f^	0.95 (0.09)	0.95 (0.08)	0^*^	-0.02, 0.01	0.709
12 months^g^	0.95 (0.08)	0.94 (0.08)	-0.01^*^	-0.02, 0.01	0.535
Waist-to-height ratio, mean (SD)					
Baseline^d^	0.81 (0.10)	0.82 (0.10)			
6 months^f^	0.68 (0.07)	0.68 (0.06)	-0.02^*^	-0.03, -0.00	0.039
12 months^g^	0.65 (0.07)	0.64 (0.07)	-0.03^*^	-0.04, -0.01	0.001
Fasting glucose, mean (SD), mmol/l					
Baseline	9.9 (4.5)	8.7 (3.5)			
6 months^h^	7.0 (2.5)	6.5 (1.9)	0.62^*^	-0.61, 1.86	0.321
12 months^d^	6.8 (2.7)	5.8 (1.4)	0.23^*^	-0.98, 1.44	0.706
Fasting insulin, median (Q1, Q3), pmol/l					
Baseline	133 (72, 218)	148 (88, 268)			
6 months^i^	43 (25, 71)	49 (30, 81)	0.87^**^	0.61, 1.23	0.417
12 months^j^	44 (25, 69)	34 (26, 72)	0.69^**^	0.48, 0.99	0.043
Fasting c-peptide, median (Q1, Q3), nmol/l					
Baseline^e^	1.2 (0.6, 1.7)	1.4 (0.8, 2.1)			
6 months^k^	0.82 (0.65, 0.99)	0.88 (0.74, 1.24)	0.92^**^	0.73, 1.16	0.477
12 months^l^	0.71 (0.53, 1.03)	0.77 (0.59, 0.95)	0.85^**^	0.67, 1.08	0.193
HOMA-IR index, median (Q1, Q3)					
Baseline	6.6 (3.5, 15.2)	7.9 (4.5, 14.7)			
6 months^i^	1.72 (0.99, 2.92)	1.68 (1.19, 2.90)	0.88^**^	0.58, 1.35	0.567
12 months^j^	1.75 (1.17, 2.63)	1.37 (0.83, 2.64)	0.66^**^	0.43, 1.03	0.068
Total cholesterol, mean (SD), mmol/l					
Baseline	4.9 (1.3)	4.8 (1.3)			
6 months^m^	3.96 (1.17)	3.75 (1.04)	-0.2^*^	-0.6, 0.2	0.349
12 months^d^	4.10 (1.20)	3.92 (0.95)	-0.1^*^	-0.4, 0.3	0.671
HDL cholesterol, mean (SD), mmol/l					
Baseline	1.2 (0.3)	1.2 (0.3)			
6 months^h^	1.21 (0.25)	1.18 (0.28)	0^#^	-0.08, 0.08	0.984
12 months^d^	1.39 (0.33)	1.34 (0.29)	-0.04^#^	-0.12, 0.04	0.340
LDL cholesterol, mean (SD), mmol/l					
Baseline	2.9 (1.2)	2.9 (1.1)			
6 months^h^	2.37 (1.06)	2.28 (0.83)	-0.09^*^	-0.39, 0.21	0.551
12 months^d^	2.39 (1.15)	2.29 (0.82)	-0.06^*^	-0.35, 0.23	0.675
Triglycerides, median (Q1, Q3), mmol/l					
Baseline	2.4 (1.9, 3.5)	2.1 (1.4, 3.0)			
6 months^h^	1.51 (1.15, 1.83)	1.53 (1.04, 1.95)	1.17^**^	0.98, 1.38	0.076
12 months^d^	1.38 (1.02, 1.66)	1.23 (0.89, 1.60)	1.13^**^	0.96, 1.34	0.146
Blood iron, mean (SD), µmol/l					
Baseline	14.5 (3.8)	13.4 (4.0)			
6 months^h^	15.4 (5.0)	13.8 (4.9)	-0.4^#^	-2.5, 1.6	0.686
12 months^n^	15.8 (5.8)	15.5 (5.1)	0.8^#^	-1.2, 2.9	0.416
Albumin, mean (SD), g/l					
Baseline	44.7 (3.2)	44.0 (3.1)			
6 months^o^	43.7 (2.6)	42.7 (3.4)	-0.3^#^	-1.5, 0.9	0.592
12 months^p^	43.4 (4.2)	42.6 (3.6)	-0.0^#^	-1.2, 1.2	0.960
Zinc, mean (SD), µmol/l					
Baseline^e^	11.9 (2.1)	11.3 (1.5)			
6 months^q^	11.1 (1.9)	10.4 (2.0)	-0.3^#^	-1.0, 0.5	0.490
12 months^r^	11.1 (2.3)	10.8 (2.0)	0.3^#^	-0.5, 1.0	0.497
Vitamin D3, mean SD), ng/l					
Baseline^s^	16.7 (7.0)	15.1 (7.1)			
6 months^t^	27.0 (10.9)	24.0 (11.7)	-1.9^#^	-5.7, 2.0	0.338
12 months^c^	26.3 (9.6)	22.3 (11.1)	-2.4^#^	-6.2, 1.3	0.201

Abbreviations: AL, alimentary limb; BPL, biliopancreatic limb; HbA1c, glycated hemoglobin; T2D, type 2 diabetes mellitus; BMI, body mass index; HOMA-IR, homeostasis model assessment of insulin resistance; HDL, high density lipoprotein; LDL, low density lipoprotein

^1^Analysis by linear regression model based on the per protocol population, unless otherwise specified

^2^T2D remission was defined as HbA1c value < 6.5% without indication for anti-diabetic medication

^3^The relative probability (“risk”) of T2D remission after long BPL-RYGB compared to long AL-RYGB was estimated with a loglinear model. If no information on T2D remission was available, no remission was assumed

^a^Data missing for 3 patients

^b^Data missing for 13 patients

^c^Data missing for 14 patients

^d^Data missing for 1 patient

^e^Data missing for 2 patients

^f^Data missing for 38 patients

^g^Data missing for 34 patients

^h^Data missing for 11 patients

^i^Data missing for 31 patients

^j^Data missing for 42 patients

^k^Data missing for 33 patients

^l^Data missing for 44 patients

^m^Data missing for 12 patients

^n^Data missing for 3 patients

^o^Data missing for 30 patients

^p^Data missing for 5 patients

^q^Data missing for 15 patients

^r^Data missing for 7 patients

^s^Data missing for 10 patients

^t^Data missing for 19 patients

^*^The negative sign indicates an advantage for the long BPL-RYGB arm

^#^The negative sign indicates a disadvantage for the long BPL-RYGB arm

^**^Dependent variable was treated logarithmically. Back-transformed values < 1 indicate an advantage for the long BPL-RYGB arm

One patient randomized to the long BPL-RYGB arm received long AL-RYGB and was analyzed as such in the per protocol analysis and in the safety set. The numbers at the 6- and 12-month follow-ups indicate how many patients provided HbA1c data.

### Primary Endpoint

At 12-month follow-up, mean HbA1c decreased from 7.8% to 6.2% in the long AL-RYGB group and from 7.3% to 5.7% in the long BPL-RYGB group. The prespecified linear regression model analysis showed a statistically significant effect of the treatment arm. Long BPL-RYGB was associated with a −0.33% lower HbA1c (Table 1). Furthermore, BMI ≥50 kg/m^2^ was associated with a −0.34% lower HbA1c in comparison to patients with BMI <50 kg/m^2^ (95% CI [−0.64, −0.04], p = 0.027). Insulin treatment was associated with 0.76% higher HbA1c (95% CI [0.47, 1.05], p <.001) and the coefficient for baseline HbA1c was 0.12 (95% CI [0.03, 0.21], p = 0.012), indicating that each percentage point of baseline HbA1c was associated with a 0.12% higher HbA1c after 12 months (Supplement 2, Table S2). In an exploratory sub-group analysis, 65 patients with baseline HbA1c > 7% and HOMA-IR > 2.5 were analyzed (n=31 standard limb lengths, n=34 changed limb lengths). Overall, the HbA1c values fell from 8.4% at baseline to 6.0% at 12 months. They were nominally superior for long BPL-RYGB, but the estimated effect was only −0.07% (95% CI [−0.49, 0.31], p = 0.71).

### Diabetes Remission and Glycemic Control

For the calculation of T2D remission rates, 2 patients from each group with a baseline HbA1c <6.5% and no antidiabetic medication were excluded from analysis. T2D remission was achieved in 64.1% of the long BPL-RYGB group and 49.2% of the long AL-RYGB group, with no significant difference between the two arms (χ^2^ (1) = 2.28, p = 0.131). The relative probability (“risk”) of T2D remission for the long BPL-RYGB group was 1.29, but not statistically significant (Table 1). Regarding fasting glucose, c-peptide and HOMA-IR, the two groups did not differ significantly. A greater decrease in fasting insulin was observed in the long BPL-RYGB group after 12 months (Table1).

### Weight Loss, Dyslipidemia, Micronutrients and Adverse Events

Comparison of the two groups for changes in body weight, BMI and waist-to-height-ratio revealed an advantage for long BPL-RYGB (Table1). Changes in waist-to-hip ratio were comparable between the two RYGB types. Furthermore, blood values for total cholesterol, HDL, LDL, and triglycerides improved similarly after both types of RYGB. Blood iron, albumin, zinc and vitamin D3 were measured as markers for malnutrition. All values remained stable or improved with postoperative supplementation, with no differences between the two groups (Table 1). Safety analysis data are reported in Supplement 2 (Tables S3-S5). Seven patients required subsequent surgical or endoscopic interventions. One patient experienced cardiac death related to a pre-existing cardiomyopathy 10 months after long AL-RYGB. Vitamin and micronutrient deficiencies were recorded in both groups, with supplementation provided after diagnosis. In the long AL-RYGB group, one case of combined nutrient deficiencies led to long-term sick leave, though intravenous substitution or parenteral nutrition was not required. No patient reported dumping symptoms.

## Discussion

In this randomized clinical trial, patients with obesity and T2D who underwent a long BPL-RYGB showed a -0.33% lower HbA1c after 12 months compared to the long AL-RYGB. This benefit could not be verified in an exploratory sub-group of patients with more pronounced diabetes. While the difference of -0.33 percentage points in HbA1c may be considered modest, it is noteworthy that the mean HbA1c of long BPL-RYGB patients after 12 months was 5.7%. The American Association of Clinical Endocrinology recommends: “optimal HbA1c is ≤6.5% or as close to normal as is safe and achievable”[13] .Since HbA1c is associated with micro- and macrovascular complications of T2D [14, 15], every decrease can lower cardiovascular risk and mortality [13] .Furthermore, 5.7% represents the lower boundary of prediabetes according to the American Diabetes Association (ADA) [16], meaning that the average long BPL-RYGB patient achieved not only diabetes remission but near-normal HbA1c levels. At baseline, the long BPL-RYGB group had a lower HbA1c (7.3% vs 7.8%) and fewer patients on insulin treatment (52% vs 60%) than the long AL-RYGB group. Since the group allocation was conducted in a randomized manner, these differences in baseline characteristics were introduced rather by chance than by bias. Nonetheless, the prespecified linear regression model analysis showed a statistically significant effect of the treatment arm. Another group analyzed optimized preoperative glucose control in bariatric patients and found that patients with lower preoperative HbA1c and insulin requirements were more likely to achieve T2D remission after RYGB. They hypothesized that these patients, who had also preoperative improvement in HbA1c might be more responsive to postoperative changes due to a better beta cell reserve [17] .The hypothetical better beta cell reserve may have contributed to the beneficial metabolic effects of the long BPL-RYGB arm in our study. 

T2D remission rates 1-5 years after RYGB vary widely in the literature with 30-63%, primarily due to differing definitions and procedural variations [18, 19]. The specific way in which RYGB was performed is often not even reported [19, 20]. In our study, which defined T2D remission in compliance with the current ADA definition, remission rates were 64.1% for long BPL-RYGB and 49.2% for long AL-RYGB, though the difference was not statistically significant. Both groups had mean HbA1c values <6.5%, highlighting once more the metabolic benefits of RYGB. Factors such as older age, higher preoperative BMI, and insulin use are known to negatively influence RYGB success in T2D treatment [21, 22]. In line with these observations, our results showed that insulin use at baseline was associated with higher HbA1c after 12 months. However, BMI ≥50 kg/m^2^ was associated with lower HbA1c, possibly reflecting a lesser influence of metabolic factors when selecting patients for MBS at higher BMI. 

The trial also found an advantage for long BPL-RYGB concerning weight loss. In a comparable RCT by Homann et al., greater weight loss was reported after a “Long-BPL-RYGB” (150 cm BPL/75 cm AL) compared to standard RYGB (75 cm BPL/150 cm AL) [23] .HbA1c was only reported after 24 and 48 months, but did not differ between groups. Contrasting our study, their primary endpoint was weight loss, not T2D improvement, and only 33% of the patients had T2D at baseline. 

Importantly, no nutritional disadvantages were observed in the long BPL-RYGB group, likely because the total length of the excluded intestine (AL+BPL) was consistent between the two surgical groups. Prior to this study, the standard RYGB limb lengths applied in our clinic were 150 / 50 cm (AL / BPL) for BMI < 50 kg/m^2^ and 170 / 80 cm (AL / BPL) for BMI > 50 kg/m^2^. Although the scientific evidence for this practice is not very strong, tailoring small bowel limb lengths to BMI is common practice among metabolic-bariatric surgeons [6, 24]. A threshold for the use of longer AL or BPL lengths is often set at BMI > 50 kg/m^2^ [24] .Systematic reviews by Orci et al. and by the ASMBS (American Society for Metabolic & Bariatric Surgery) also state that patients with BMI ≥ 50 kg/m^2^ might benefit from longer limb lengths [8, 25] .When planning this study, we decided to maintain our usual practice and merely switched the lengths of AL and BPL in the long BPL group. Therefore, with the exception of patients with BMI ≥ 50 kg/m^2^, the combined length of AL+BPL was 200 cm as recommended by IFSO [7]. Thus, the positive effects of long BPL-RYGB on T2D and weight loss were likely not induced by malabsorption but by other mechanisms. An observational study that included only patients with obesity and T2D reported increased weight loss 24 months and T2D remission 36 months after application of a long BPL (200 cm) in comparison to a short BPL (50-90 cm) [26] .The same group around Mariana Monteiro had conducted a cadaver study in advance, characterizing the distribution of incretin-producing cells along the human small intestine [27] .They observed a significant increase of glucagon-like peptide-1 (GLP-1)-producing L-cells 200 cm distal the ligament of Treitz, which is why they assigned this length to their “long BPL”. The accelerated delivery of nutrients to the L-cells-carrying part of the small intestine is assumed to lead to the known postprandial increase of plasma GLP-1 after RYGB and thus improved glycemic control [28, 29]. Significantly higher plasma GLP-1 levels after RYGB with long BPL in comparison to short BPL have also been reported by Monteiro and co-workers [30]. In that study, they also measured fasting insulin and c-peptide, which were not different. Although in our study, fasting insulin reduction was greater in the long BPL-RYGB group, c-peptide and HOMA-IR did not differ. Incretin levels were not measured. Therefore, this study cannot contribute further insights to the question how BPL length effects gut hormone secretion. 

A limitation of this study is that we were unable to randomize the initially foreseen conservative therapy group and the study is not powered for the comparison of the two surgical arms. Moreover, the evaluation of micronutrients to assess for nutritional deficiencies reported here is limited. Future follow-up reports will need to include e.g. vitamin B12 and markers of calcium homeostasis, such as bone specific alkaline phosphatase and parathormone to verify that no differences in micronutrient status exists between groups. Future follow-ups would also have to include a more specific assessment of post-prandial hyperinsulinemic hypoglycemia. It is a very important and pleasing result that up to 12 months postoperatively none of the study participants reported dumping symptoms. However, future specific testing will provide more information on the possible influence of a long BPL on dumping syndrome. A further limitation is the comparatively low HbA1c values at baseline. Finally, the relatively short-term results meaning that T2D relapse, weight regain and nutritional status could not be assessed in the long term after RYGB [22, 31]. Therefore, follow-up of the surgical patients is ongoing and will be reported in the future.

## Conclusion

This RCT suggests that RYGB with a longer BPL is associated with greater improvements in HbA1c and weight loss without showing higher risk for nutritional complications in comparison to RYGB with longer AL. However, diabetes remission rates did not differ significantly between procedures at 12 months. These findings indicate that modification of limb lengths may influence selected metabolic parameters, while its impact on categorical diabetes remission may require longer follow-up or larger, adequately powered studies.

## Supplementary Information

Below is the link to the electronic supplementary material.

Supplementary Material 1 (DOCX 32.9 KB)

Supplementary Material 2 (PDF 631 KB)

## Data Availability

The data may be shared in de-identified form if appropriate permits are sought and obtained.
