# Supplementary material for: Effect of RYGB Limb Lengths on HbA1c in Patients with Obesity and Type 2 Diabetes
Source: Obes Surg. 2026 Apr 21;36(6):2976–86. doi: 10.1007/s11695-026-08690-6 (PMC13249693; doi:10.1007/s11695-026-08690-6)
Supplement: Supplementary file 1 — Supplementary Material 1 (DOCX 32.9 KB) [file 11695_2026_8690_MOESM1_ESM.docx]

**Supplement 2**

Effect of RYGB Limb Lengths on HbA1c in Patients with Obesity and Type 2 Diabetes.

**TableS1.** List of *R* software packages used for statistical analyses

**Table S2.** Results of the primary endpoint analysis using the prespecified linear regression model

**Table S3.** Severe adverse events recorded through 12 months post-RYGB

**Table S4.** Adverse events recorded through 12 months post-RYGB

**Table S5.** Additionally performed procedures during RYGB surgery (not classified as AEs)

**Table S1. List of *R* software packages used for statistical analyses**

• R (version 4.3.2; R Core Team, 2023)

• lme4 (version 1.1.35.2; Bates D et al., 2015)

• Matrix (version 1.6.5; Bates D et al., 2024)

• infer (version 1.0.7; Couch SP et al., 2021)

• Rcpp (version 1.0.12; Eddelbuettel D et al., 2024)

• Gmisc (version 3.0.3; Gordon M, 2023)

• htmlTable (version 2.4.2; Gordon M et al., 2023)

• lubridate (version 1.9.3; Grolemund G, Wickham H, 2011)

• glue (version 1.7.0; Hester J, Bryan J, 2024)

• huxtable (version 5.5.6; Hugh-Jones D, 2024)

• ggdist (version 3.3.2; Kay M, 2024)

• CTCLmetasurg (version 0.0.10; Koehler N, 2024)

• lmerTest (version 3.1.3; Kuznetsova A et al., 2017)

• emmeans (version 1.10.1; Lenth R, 2024)

• report (version 0.5.8; Makowski D et al., 2023)

• tibble (version 3.2.1; Müller K, Wickham H, 2023)

• mdthemes (version 0.1.0; Neitmann T, 2020)

• patchwork (version 1.2.0; Pedersen T, 2024)

• broom (version 1.0.5; Robinson D et al., 2023)

• gtsummary (version 1.7.2; Sjoberg D et al., 2021)

• ggplot2 (version 3.5.0; Wickham H, 2016)

• forcats (version 1.0.0; Wickham H, 2023)

• stringr (version 1.5.1; Wickham H, 2023)

• tidyverse (version 2.0.0; Wickham H et al., 2019)

• dplyr (version 1.1.4; Wickham H et al., 2023)

• purrr (version 1.0.2; Wickham H, Henry L, 2023)

• readr (version 2.1.5; Wickham H et al., 2024)

• tidyr (version 1.3.1; Wickham H et al., 2024)

• kableExtra (version 1.4.0; Zhu H, 2024)

**Table S2. Results of the primary endpoint analysis using the prespecified linear regression model**

| **Characteristic** | **Beta** | **95% CI** | **p-value** |
| --- | --- | --- | --- |
| (Intercept) | 4.9 | 4.2, 5.6 | <0.001 |
| Arm |  |  |  |
| long AL-RYGB | -- | -- |  |
| long BPL-RYGB | -0.33 | -0.60, -0.06 | 0.018 |
| HbA1C (baseline) | 0.12 | 0.03, 0.21 | 0.012 |
| Sex |  |  |  |
| Female | -- | -- |  |
| Male | -0.05 | -0.34, 0.23 | 0.704 |
| BMI categorical (baseline) |  |  |  |
| BMI < 50 kg/m2 | -- | -- |  |
| BMI ≥ 50 kg/m2 | -0.34 | -0.64, -0.04 | 0.027 |
| Insulin treatment (baseline) |  |  |  |
| No insulin treatment | -- | -- |  |
| Insulin treatment | 0.76 | 0.47, 1.0 | <0.001 |

Abbreviations: CI, confidence interval; AL, alimentary limb; RYGB, Roux-en-Y gastric bypass; BPL, biliopancreatic limb; HbA1c, glycated hemoglobin; BMI, body mass index.

**Safety Analysis**

Safety analysis was conducted on all patients who underwent either long AL-RYGB or long BPL-RYGB. Patients were analyzed according to the treatment they actually received. Patients who started the trial but later withdrew or were lost to follow-up were included in the analysis until their discontinuation.

**Table S3. Severe adverse events recorded through 12 months post-RYGB**

| **Arm** | **SAE Term** | **SAE No.** |
| --- | --- | --- |
| **long BPL-RYGB** | Intraabdominal dislocation of drain tube^a^ | 1 |
| **long BPL-RYGB** | Anastomotic leakage^b^ | 1 |
| **long AL-RYGB** | Cholecystolithiasis | 1 |
| **long BPL-RYGB** | Cataract | 1 |
| **long BPL-RYGB** | Hepatic encephalopathy^c^ | 1 |
| **long BPL-RYGB** | Hepatic encephalopathy^c^ | 2 |
| **long BPL-RYGB** | Diagnosis of carotid stenosis | 1 |
| **long AL-RYGB** | Anastomotic bleeding^d^ | 1 |
| **long AL-RYGB** | Diagnosis of renal cell carcinoma | 1 |
| **long AL-RYGB** | Resection of renal cell carcinoma | 2 |
| **long AL-RYGB** | Combined nutrient deficiency leading to long term sick leave | 3 |
| **long BPL-RYGB** | Perforation of the Colon ascendens^e^ | 1 |
| **long BPL-RYGB** | Meningitis^e^ | 1 |
| **long AL-RYGB** | Cardiac death^f^ | 1 |

Adverse events that resulted in death, were life-threatening, required hospitalization or prolongation of hospitalization or resulted in persistent or significant disability / incapacity were recorded as severe adverse events (SAE).

In total, 14 SAEs were recorded in 11 patients.

^a^An accidental dislocation of an abdominal drain tube into the abdominal cavity at postoperative day 4 led to revisional laparoscopy, followed by a then uncomplicated postoperative course.

^b^After an initially uncomplicated postoperative course, one patient was readmitted to the hospital 44 days after surgery with an acute abdomen. Emergency revisional laparoscopy revealed a local peritonitis due to a covered leakage from the jejunojejunostomy. After laparoscopic lavage and drainage the patient recovered rapidly.

^c^One patient suffered from two episodes of hepatic encephalopathy requiring hospitalization. The first episode was triggered by a gastroenteritis, the second by dehydration due to diuretic treatment of recurrent ascites. The patient had a known liver cirrhosis (Child B) and metabolic surgery was scheduled to achieve fitness for liver transplantation wait listing. Fourteen months after RYGB, the patient was successfully transplanted and has a good organ function ever since.

^d^One patient, who was under therapeutic anticoagulation, had a bleeding of the gastrojejunostomy on postoperative day 5 which was successfully treated endoscopically.

^e^These two cases were not included in the final study analysis, since the patients withdrew their consent for further participation. One patient suffered from an acute perforation of the ascending colon three months after long BPL-RYGB and an open hemicolectomy with colonostomy had to be performed. The other patient was admitted to another hospital because of a meningitis 11 months after long BPL-RYGB.

^f^There was one case of death in a patient related to a pre-known dilatative cardiomyopathy 10 months after long AL-RYGB. Since the death occurred prior the 12-months study visit, this patient was also not included in the final analysis.

**Table S4. Adverse events recorded through 12 months post-RYGB**

| **Visit** | **Adverse event** | **long AL-RYGB** | **long BPL-RYGB** | **Total** |
| --- | --- | --- | --- | --- |
| **1-month** | Impaired wound healing | 1 | 2 | 3 |
|  | Dizziness, Circulation | 0 | 3 | 3 |
|  | Pain (abdominal, back) | 1 | 1 | 2 |
|  | Magnesium deficiency | 1 | 0 | 1 |
|  | Other | 1 | 1 | 2 |
| **3-month** | Zinc deficiency | 5 | 6 | 11 |
|  | Hypocortisolism | 3 | 2 | 5 |
|  | Iron deficiency | 3 | 2 | 5 |
|  | Pain (abdominal, back) | 2 | 1 | 3 |
|  | Calcium deficiency | 1 | 1 | 2 |
|  | Magnesium deficiency | 0 | 2 | 2 |
|  | Folic acid deficiency | 1 | 0 | 1 |
|  | Albumin deficiency | 0 | 1 | 1 |
|  | Diarrhea | 0 | 1 | 1 |
|  | Dizziness, Circulation | 0 | 1 | 1 |
|  | Hypokalemia | 0 | 1 | 1 |
|  | Total protein deficiency | 0 | 1 | 1 |
|  | Vitamin D3 deficiency | 0 | 1 | 1 |
|  | Other | 0 | 4 | 4 |
| **6-month** | Zinc deficiency | 34 | 39 | 73 |
|  | Iron deficiency | 5 | 8 | 13 |
|  | Hypocortisolism | 7 | 5 | 12 |
|  | Total protein deficiency | 5 | 7 | 12 |
|  | Magnesium deficiency | 4 | 7 | 11 |
|  | Hypokalemia | 5 | 2 | 7 |
|  | Ferritin deficiency | 3 | 3 | 6 |
|  | Folic acid deficiency | 2 | 3 | 5 |
|  | Albumin deficiency | 1 | 3 | 4 |
|  | Calcium deficiency | 2 | 2 | 4 |
|  | Pain (abdominal, back) | 2 | 2 | 4 |

(Continued)

**Table S4. Adverse events recorded through 12 months post-RYGB (continued)**

| **Visit** | **Adverse event** | **long AL-RYGB** | | **long BPL-RYGB** | **Total** |
| --- | --- | --- | --- | --- | --- |
| **6-month** | Vitamin D3 deficiency | 1 | 2 | | 3 |
|  | Dizziness, Circulation | 0 | 3 | | 3 |
|  | Diarrhea | 1 | 1 | | 2 |
|  | Other | 4 | 4 | | 8 |
| **12-month** | Zinc deficiency | 33 | 33 | | 66 |
|  | Total protein deficiency | 8 | 10 | | 18 |
|  | Hypocortisolism | 5 | 7 | | 12 |
|  | Iron deficiency | 5 | 5 | | 10 |
|  | Folic acid deficiency | 3 | 3 | | 6 |
|  | Hypokalemia | 2 | 4 | | 6 |
|  | Calcium deficiency | 2 | 3 | | 5 |
|  | Ferritin deficiency | 3 | 1 | | 4 |
|  | Magnesium deficiency | 2 | 2 | | 4 |
|  | Albumin deficiency | 1 | 2 | | 3 |
|  | Dizziness, Circulation | 1 | 1 | | 2 |
|  | Other | 2 | 2 | | 4 |
| **Total** | NA | 162 | 195 | | 357 |

Adverse events (AEs) were recorded over the course of the study regardless of whether they were related to the intervention or not. They were listed at the time point of the study they were reported to the study personnel. Persisting events that were reported repeatedly are also listed repeatedly.

**Table S5. Additionally performed procedures during RYGB surgery (not classified as AEs)**

| **Procedure** | **long AL-RYGB** | **long BPL-RYGB** | **Total** |
| --- | --- | --- | --- |
| Adhesiolysis | 13 | 24 | 37 |
| Hiatal hernia repair | 13 | 16 | 29 |
| Liver biopsy | 4 | 5 | 9 |
| Local excision of tumorous tissue^a^ | 2 | 5 | 7 |
| Umbilical hernia repair | 1 | 4 | 5 |
| Cholecystectomy | 1 | 3 | 4 |
| Resection of omentum | 1 | 0 | 1 |
| Abdominal wall hernia repair | 0 | 1 | 1 |
| Total | 35 | 58 | 93 |

^a^In 7 cases, tumorous tissues suspicious for malignancy were resected during RYGB surgery. Histological examination revealed benign diagnoses in 4 cases and low-risk gastrointestinal stromal tumors in 3 cases (all in the long BPL-RYGB group) that did not require further treatment or diagnostics.
