# Supplementary material for: Effect of RYGB Limb Lengths on HbA1c in Patients with Obesity and Type 2 Diabetes
Source: Obes Surg. 2026 Apr 21;36(6):2976–86. doi: 10.1007/s11695-026-08690-6 (PMC13249693; doi:10.1007/s11695-026-08690-6)
Supplement: Supplementary file 2 — Supplementary Material 2 (PDF 631 KB) [file 11695_2026_8690_MOESM2_ESM.pdf]

Supplement 1

Effect of RYGB Limb Lengths on HbA1c in Patients with Obesity and Type 2 Diabetes.

The trial protocol is provided in its original approved version; statements therein reflect the wording of the protocol document. This applies in particular to page 11, section 1.2.1, where RYGB is described as an irreversible procedure. For clarity, RYGB is generally considered reversible in principle.

[redacted]

Trial Protocol

Metabolic Surgery for Type 2 Diabetes within BMI range of 27 to 60 kg/m^2

MetaSurg-Study; **(Meta**bolic **Surg**ery)

Coordinating investigator **Biometry / Project Management**

[redacted]

Trial protocol:	Version: Final 7.0	Date: 18.11.2021
incl. Amendment 01:	Version: Final 1.0	Date: 22.01.2016
incl. Amendment 02:	Version: Final 1.0	Date: 29.06.2016
incl. Amendment 03:	Version: Final 1.0	Date: 19.12.2016
incl. Amendment 04:	Version: Final 1.0	Date: 08.02.2019
incl. Amendment 05:	Version: Final 1.0	Date: 28.12.2019
incl. Amendment 06:	Version: Final 1.0	Date: 26.11.2020
incl. Amendment 06:	Version: Final 1.0	Date: 26.11.2020

Universal Trial Number (UTN): [redacted]

The information provided in this document is confidential and is not to be circulated to third parties.

© Version final 7.0 valid as of 22.10.2021

Print: []

[redacted]

Table of Contents

page

GENERAL INFORMATION ... 5

Responsible Parties ... 5
Synopsis ... 7
Schedule of Assessments and Procedures ... 9
Flow Chart ... 10

1 RATIONALE ... 11

1.1 Medical Background ... 11
1.2 Rationale ... 11
1.3 Risk-Benefit Considerations ... 13

2 OBJECTIVES ... 13

2.1 Primary Objective ... 13
2.2 Secondary Objectives ... 13

3 TRIAL DESIGN AND DESCRIPTION ... 14

3.1 Trial Design ... 14
3.2 Requirements at the Trial Sites Regarding Personnel and Equipment ... 14
3.3 Trial Sites and Number of Trial Subjects ... 14
3.4 Expected Duration of Trial ... 15
3.5 Premature Termination of the Trial ... 15

4 TRIAL SUBJECTS ... 15

4.1 Inclusion Criteria ... 15
4.2 Exclusion Criteria ... 15

5 TRIAL INTERVENTION ... 16

5.1 Standard RYGB (stRYGB) group and Changed limb length RYGB (cll RYGB) group ... 16
5.2 Observational Control (Medical treatment group) ... 17

6 INDIVIDUAL TRIAL PROCEDURES ... 17

6.1 Screening ... 17
6.2 Patient Information and Informed Consent ... 17
6.3 Enrolment in the Trial (Baseline-Visit) and Randomisation ... 18
6.4 Description of further Study Visits ... 19
6.5 Premature Termination of the Therapy or Follow-up ... 20

7 METHODS OF DIAGNOSTICS AND DATA SAMPLING ... 21

8 ADVERSE EVENTS (AE/SAE) ... 22

8.1 Adverse Events (AE) ... 22
8.2 Serious Adverse Events (SAE) ... 22
8.3 Safety Analyses ... 22
8.4 Concomitant disease ... 22
8.5 Therapeutic Procedures ... 23

9 BIOMETRY ... 23

9.1 Randomisation ... 23
9.2 Outcomes ... 23
9.3 Statistical Description of the trial hypothesis ... 24
9.4 Sample Size Discussion ... 25

9.5 Statistical Methods ... 25
9.6 Interim Analysis ... 25
9.7 Final Analysis ... 25

10 ETHICAL, LEGAL AND ADMINISTRATIVE ASPECTS ... 26

10.1 GCP-Statement ... 26
10.2 Submission ... 26
10.3 Protocol Amendments ... 26

11 DOCUMENTATION ... 26

11.1 Case Report Forms (CRF) ... 26
11.2 Data Management ... 27
11.3 Archiving ... 27

12 SUPERVISION OF THE CLINICAL TRIAL ... 28

12.1 Access to Source Data ... 28
12.2 Monitoring ... 28
12.3 Independent Supervision of the Trial ... 28

13 DATA PROTECTION AND CONFIDENTIALITY ... 28

14 ADMINISTRATIVE AGREEMENTS ... 29

14.1 Adherence to the Protocol ... 29
14.2 Funding and Insurance ... 29
14.3 Publication Policy and Registration ... 30

15 CONCOMITANT SCIENTIFIC PROJECT ... 30

15.1 LiMAX-Test ... 30
15.2 MRI (brain and spinal cord) and cognitive function ... 31

16 PROTOCOL SIGNATURES ... 32

17 PROTOCOL AGREEMENT ... 32

18 APPENDIX ... 33

18.1 Classification of Adverse Events ... 33
18.2 Acronyms ... 34

19 REFERENCES ... 35

GENERAL INFORMATION

Responsible Parties

Coordinating investigator(s)	[redacted]
Project Management	[redacted]
Medical consultant(s)	[redacted]
Data Management	[redacted]
Biometry	[redacted]
Monitoring	[redacted]
Funding	[redacted]

	[redacted]
Trial Steering Committee	

Synopsis

Title of the trial	Metabolic Surgery for Type 2 Diabetes within BMI range of 27 to 60 kg/m^2
Acronym	**MetaSurg**-Study; (Metabolic Surgery)
Indication	**Type 2 Diabetes mellitus**
Primary goal of the trial	In this project, we will investigate the short and mid-term effects of standard Roux-en-Y gastric bypass (stRYGB) and changed limb length RYGB (cllRYGB) in Type 2 diabetes (T2DM) patients in the context of metabolic surgery.
Trial design	Open prospective randomized parallel group trial with an additional observational group
Trial population	Key inclusion criteria:<br>• BMI ≥27 to ≤60 kg/m^2<br>• T2DM<br>– for patients BMI ≥35 kg/m^2: any T2DM<br>– for patients BMI <35 kg/m^2:<br>– unsatisfactory medical non-insulin treatment (multiple medications, HbA1c >6.5%, hypo- or hyperglycaemias), or<br>– planned insulin treatment or ongoing insulin treatment<br>• males and females, age ≥18 years<br>• written informed consent<br>Key exclusion criteria:<br>• any chronic inflammatory or malignant disease<br>• type 1 diabetes<br>• peptic ulcer<br>• contraindication for general anaesthesia<br>• drug or alcohol abuse<br>• untreated thyroid dysfunction<br>• pregnancy or breast feeding woman<br>• expected non-compliance<br>participation in other interventional trial requiring insurance
Sample size	To be assessed for eligibility: n = 1500<br>To be allocated to trial: n = 160 (plus up to 20patients in respect of Amandment5 for additional neurocognitive examinations)<br>To be analysed: n = 140 (plus up to 20patients in respect of Amandment5 for additional neurocognitive examinations)
Therapy	Experimental intervention: standard RYGB and changed limb length RYGB<br>Control intervention / reference test: control arm with standard medical diabetes treatment in patients who decline surgical treatment or where health insurance rejects the application for it
Primary end point	Primary efficacy endpoint: HbA1c value after 1 year
Secondary end points	Main secondary efficacy endpoints:<br>for the two surgical arms: T2DM remission rate after 1 year, definition of T2DM remission: HbA1c <6.5% without any antidiabetic medication

Biometry	Efficacy: Comparison of the interventions and control with respect to the HbA1c values after 1 year<br>Description of the primary efficacy analysis and population:<br>The primary endpoint will be analysed using ANCOVA for the HbA1c levels, with the baseline value as covariate and the arm along with stratification variables as factors. Closed testing procedures will be used for the multiple comparisons between arms.<br>Safety: Descriptive statistics will be provided for procedure/device related adverse events by trial arm.<br>Secondary endpoint: Diabetes remission rates will be compared using a chi-squared-test providing an estimate with 95% confidence interval for the relative probability ("risk") of remission rates.
Trial Duration	**Individual**: Duration of intervention per patient: 12 months<br>**Trial**: Duration of the entire trial (months): 86 months<br>Recruitment period (months): 62 months<br>Data analysis and publication: 6 months after the last visit of the last patient

Schedule of Assessments and Procedures

Visit Nr.	Visit 1 (Baseline)	Visit 2	Visit 3	Visit 4	Visit 5	Visit 6	Visit 7	Visit 8
**Time, month** [1]	0	1 (±1 week)	3 (±1 week)	6 (±4 weeks)	12 (±2 month)	36 (±3 month)	60 (±5 month)	120 (±10 month)
Age/gender	X							
Informed consent	X							
In-/exclusion criteria	X							
Randomisation	X							
Medical history	X							
Concomitant diseases	X	X	X	X	X	X	X	X
Concomitant medication	X	X	X	X	X	X	X	X
Pregnancy test	X							
Vital signs (systolic & diastolic blood pressure, pulse etc.)	X	X	X	X	X	X	X	X
Diet protocol	X				X			
Questionnaire well-being (SF 36) and physical activity (IPAQ) and cognitive function	X			X	X		X	X
Fasting blood sample HbA$_{1c}$, glucose, insulin, adipokines, lipids, parameters of inflammation, hematology, biochemistry etc.	X	X	X	X	X	X	X	X
BIA measurement	X			X	X	X	X	X
Intima-media-thickness (IMT)	X			X	X			
Ultrasound, fibroscan	X			X	X			
ELF-test	X			X	X			
MRI (visceral fat, liver size, brain and spinal cord)	X			X	X			
LiMAx-Test (liver function)	before and after praeoperative diet		X	X				
Indirect calorimetry	X			X	X			
adverse events	X	X	X	X	X			

[1] The timing of the follow-up visits based on the date of the intervention for patients in the intervention groups and based on the date of the baseline examination for control patients.

Flow Chart

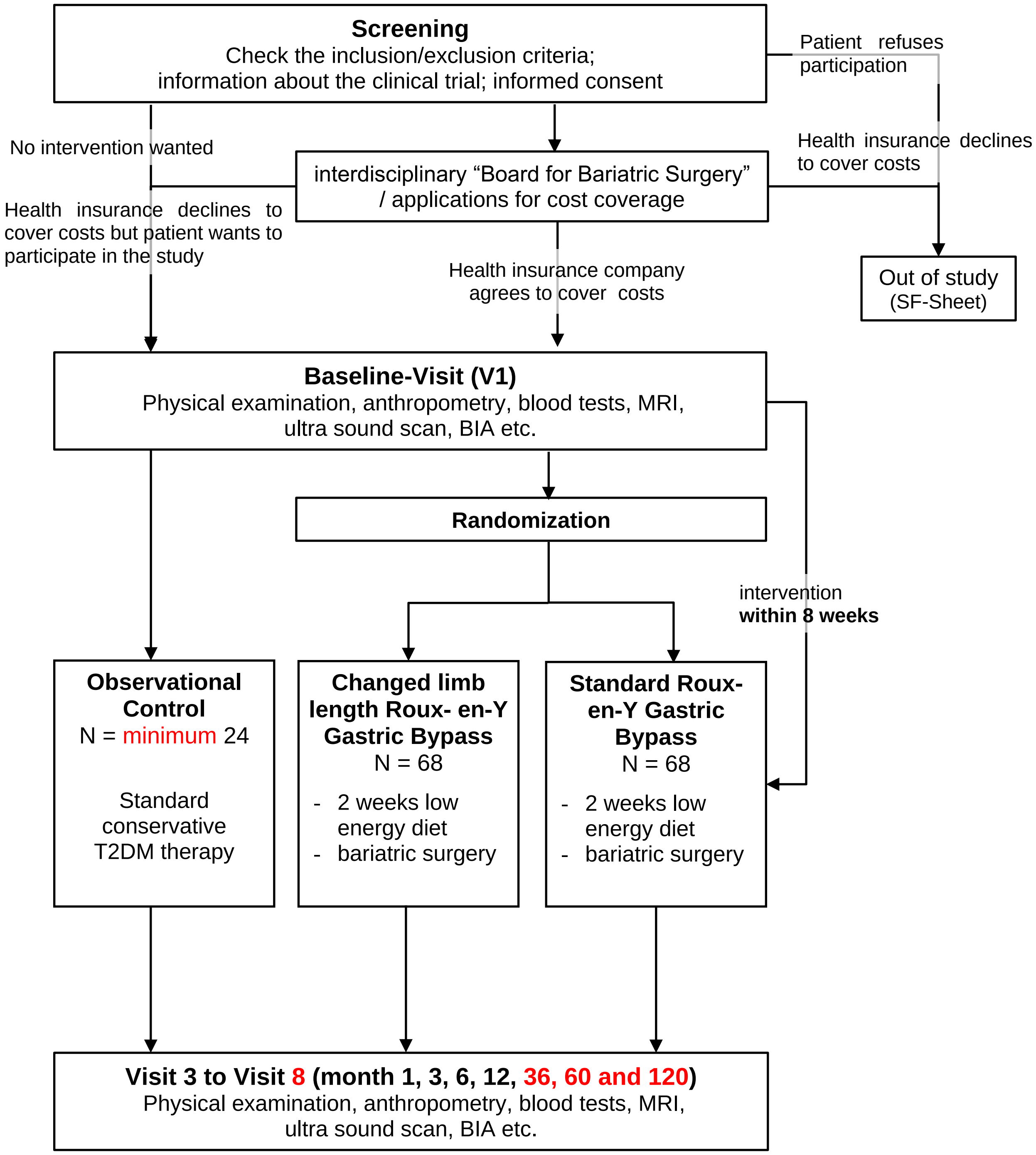
Screening
Check the inclusion/exclusion criteria;
information about the clinical trial; informed consent
Patient refuses participation
No intervention wanted
interdisciplinary "Board for Bariatric Surgery" / applications for cost coverage
Health insurance declines to cover costs
Health insurance declines to cover costs but patient wants to participate in the study
Health insurance company agrees to cover costs
Out of study
(SF-Sheet)
Baseline-Visit (V1)
Physical examination, anthropometry, blood tests, MRI, ultra sound scan, BIA etc.
Randomization
intervention
within 8 weeks
Observational Control
N = minimum 24
Standard conservative T2DM therapy
Changed limb length Roux- en-Y Gastric Bypass
N = 68
- 2 weeks low energy diet
- bariatric surgery
Standard Roux-en-Y Gastric Bypass
N = 68
- 2 weeks low energy diet
- bariatric surgery
Visit 3 to Visit 8 (month 1, 3, 6, 12, 36, 60 and 120)
Physical examination, anthropometry, blood tests, MRI, ultra sound scan, BIA etc.

1 RATIONALE

1.1 Medical Background

T2DM is a major burden on public health due to its high prevalence and the association with severe co-morbidities. Good glycaemic control decreases long-term morbidity and mortality. However, HbA1c levels are unsatisfactory high for many patients. A certain number of prospective trials demonstrated that bariatric procedures (mostly RYGB) can address this problem.

So far, standard RYGB is the operation of choice for most surgeons in metabolic surgery.

However, standardised evidence based limb length recommendations do not exist for the RYGB in bariatric or metabolic surgery.

Due to a longer biliopancreatic limb, the mini-bypass might be better for both weight loss and T2DM improvement, though randomized trials are lacking.

Mimicking this effect, it may be simplest merely to exchange the limb lengths, favouring a long biliopancreatic and short alimentary limb.

Here with a Roux-en-Y reconstruction we do not have the risk of bile reflux into the pouch (and unclear GERD and cancer risk) that are seen with the mini-bypass.

The MetaSurg Study is an open, randomized controlled trial comparing standard RYGB with RYGB with changed limb length and a control arm with standard medical treatment in T2DM patients in a BMI range of 27 to 60 kg/m2. The primary endpoint is HbA1c improvement after 1 year. The key secondary endpoint for the surgical arms is the diabetes remission rate after 1 year. Diabetes remission is defined as follows: HbA1c <6.5% without any anti-diabetic medication. 68 patients per intervention arm are planned plus 24 patients for the control arm (to be analysed: 60:60:20).

Patients will have a study follow-up of 1 year and further secondary endpoints are weight loss, visceral fat mass, therapy costs and need of medication for other co-morbidities. At our centre, there will be a lifelong follow up with continued data collection.

We expect RYGB with changed limb length to be superior to the standard RYGB with lowest HbA1c levels, a higher diabetes remission rate, relevant weight loss, and higher remission rate of obesity related co-morbidities.

1.2 Rationale

1.2.1 Hypothesis and Experimental Aspects of the Clinical Trial

T2DM and obesity are major societal burdens that are expected to increase with time. For many patients with known and medically treated T2DM, the HbA1c levels are unsatisfactorily high. Moreover, there are a number of patients suffering from hypo- or hyperglycaemic episodes.

In morbidly obese patients, bariatric surgery is a widely accepted therapy option. So far, gastric bypass is the so called “gold standard”. Besides long lasting weight loss, there are well known metabolic benefits.

Bypassing the proximal small intestine and food restriction following RYGB has been shown to improve glucose metabolism dramatically within days, prior to weight loss in obese patients with T2DM. Depending on the definition, remission rates of up to 80% are reported. However, RYGB is a major surgical procedure, related to relevant morbidity and mortality and is irreversible. In a meta-analysis Buchwald et al reported a 30-day mortality after RYGB of 0.5%. [1]

The Roux-en-Y gastric bypass can be considered to be the gold standard for metabolic surgery. However, there is no definition of the optimal limb length in either bariatric or metabolic surgery. Some data favour a longer biliopancreatic limb over a longer alimentary limb. Nergaard at al. showed greater weight loss for the longer biliopancreatic limb and also metabolic improvement; however, the metabolic effects require further studies due to the study design. [9]

The reabsorption of bile acids has an independent positive metabolic effect. Obese patients are known to have lower serum bile acid levels. Following bypass surgery, serum bile acids increase and they are most likely an independent factor for metabolic improvement.

They act on different pathways and influence glucose metabolism, insulin resistance, lipid metabolism, gut hormones, gut microbiota etc. in a beneficial way for the patient. [5-8]

Most likely the mini-bypass functions that way – having a long biliopancreatic limb. However, with a Roux-en-Y reconstruction we do not have the risk of bile reflux into the pouch (and unclear GERD and cancer risk).

In our trial, we intend to investigate the efficacy and safety of the stRYGB and cllRYGB compared to standard medical treatment in the short and midterm on patients suffering T2DM.

The Bariatric Surgery Section is well prepared for such study; we are currently performing a prospective randomized trial on glucose metabolism with an exercise intervention following bypass surgery (GluMBSE, K74-1). From 2013 we performed little more than 100 bariatric operations per year, mostly RYGB and in January 2014 we became a certified "Competence Center for Bariatric Surgery".

A number of outpatient endocrinologists / diabeticians have agreed to participate in the study.

Metabolic surgery (surgery mainly for T2DM) is continually gaining acceptance. There are only few data from prospective trials, but all recent reviews/meta-analyses indicate that metabolic surgery is beneficial. However, due to the lack of (long term) data, no conclusive statement is possible and more studies are required. [2] Consequently, in the German guideline for Bariatric Surgery metabolic surgery is recommended for T2DM in patients with a BMI <35 kg/m^2 preferably within studies. [3]

There is a lack of studies investigating the limb length of RYGB with respect to best metabolic changes.

In this prospective randomized trial we want to compare cllRYGB with the current gold standard stRYGB for metabolic surgery and compare these to standard non-surgical care.

We aim to:

1. Compare the efficacy of medical treatment, cllRYGB and stRYGB regarding HbA1c change
2. Compare cllRYGB and stRYGB regarding diabetes remission.
3. Implement limb length recommendations for the RYGB into guidelines.
4. Demonstrate the efficacy of metabolic surgery. As mentioned, several small trials suggest that metabolic surgery is beneficial. So far, metabolic surgery is only recommended when performed within studies. We hope to increase the value of existing data to be implemented in new guidelines.
5. By having a long term follow up at our centre, we hope to collect and analyse important long term data later on.
6. We want to prove that metabolic surgery is beneficial to the patients not only for glycaemic control. Since a sufficient weight loss will be achieved in most patients we expect other obesity associated comorbidities (such as hypertension, GERD, sleep-apnea-sydrome, fatty liver, NASH etc.) to improve.

1.3 Risk-Benefit Considerations

The trial will be conducted in accordance with the Guidelines for Good Clinical Practice (GCP). Prior to its start, the trial has to be approved by the Ethical Committee at the University of [redacted]

The study will be fully explained to participating patients and inclusion will not be initiated prior to obtained written consent. Patients can withdraw at any point without any disadvantage.

All treatment activities within the study (such as operations (RYGB), endoscopy (routine [redacted] prior RYGB), oral or subcutaneous antidiabetic medication) are standard [redacted] for the population treated and thus covered by the medical insurance.

All follow-up examinations are without any risk and blood samples will only be taken when routine blood will be taken anyway.

Compared to the risk of (poor glycaemic control and) developing diabetes complications, the risks of metabolic surgery are low. However, there are no long term data.

All follow-up examinations are without any risk and blood samples will not be taken for the study alone.

The benefits expected from this trial (better T2DM control, substantial weight loss) clearly outweigh the slight risks.

Participants of the control group will benefit from the much more intensive “Bariatric follow up”, what includes an intensive questioning of eating behaviours, extended laboratory investigations (vitamins, trace elements and supplementation if indicated) and investigations such as resting energy expenditure or body fat mass etc..

2 OBJECTIVES

2.1 Primary Objective

<u>Primary efficacy endpoint</u>:

- HbA1c values after 1 year

2.2 Secondary Objectives

<u>Main secondary efficacy endpoint:</u>

- T2DM remission rate after 1 year
  Definition T2DM remission: HbA1c <6.5% without indication for anti-diabetic medication
- Weight loss
- General wellbeing (SF-36 questionnaire, IPAQ) and cognitive function (TMT and MST task)
- Anthropometry (Body weight, BMI, WHR (waist to hip ratio), Body fat mass (BIA))
- Clinical parameters (fasting glucose, fasting insulin, fasting c-peptide, HOMA-IR index, Serum lipids)

<u>Further investigated parameters:</u>

- MRI (liver, subcutaneous and visceral fat mass, brain and spinal cord: inflammation, size general and –grey matter)
- Intima-media-thickness (IMT) A. carotis com.
- Liver volume (MRI), function and morphology
- LiMAx test (functional liver test)
- Indirect calorimetry
- Serum concentration of bile acids, GLP1, PYY, GIP, ghrelin (total and active), obestatin, leptin, leptin BP, adiponectin, chemerin, fetuin-A, Nampt, RBP4, Vaspin, OBBP, betatrophin (presently favored to be examined; extra serum will be frozen and stored for potentially new future parameters)
- Inflammatory markers: CrP, WBC, IL-6, TNFα
- Average caloric intake per day

3 TRIAL DESIGN AND DESCRIPTION

3.1 Trial Design

Open prospective randomized parallel group trial with an additional observational control arm.

The patients in the two surgical arms will not be informed as to which version of the operation they received until completion of the trial whereas, clearly, the control arm cannot be blinded with regard to treatment. If logistically feasible, study visits will be performed by personnel unaware of which surgical procedure was performed.

3.2 Requirements at the Trial Sites Regarding Personnel and Equipment

The study management is experienced and trained in clinical trials. Similar studies (such as the IFB STEN or GluMBSE study) are currently ongoing with overlapping personnel.

All examinations (taking of blood samples, BIA measurement, MRI, indirect calorimetry, ultrasound etc.) will be performed by trained (and experienced) personnel following a detailed working instruction. All planned examinations were performed frequently in the past and are part of the daily routine. None of the above examinations is new or has to be established.

The equipment needed for the clinical trial part is available in the clinic (such as MRI, BIA equipment, indirect calorimetry etc.).

The study invasive procedure (RYGB) is well established and part of the clinical practice at our hospital.

For the scientific part, the fully equipped laboratory capacity of the Departments of Medicine, Bariatric Surgery and Laboratory Medicine will be used.

3.3 Trial Sites and Number of Trial Subjects

The study is a single centre trial.

<u>To be assessed for eligibility:</u>	n = 1500
<u>To be allocated to trial:</u>	n = 160
<u>To be analysed:</u>	n = 140

3.4 Expected Duration of Trial

Individual trial duration:
Duration of intervention per patient: 1 year

Complete trial duration:

Duration of the entire trial (months):	86
Recruitment period (months):	74
Data analysis and publication:	6 months after the last visit of the last patient

3.5 Premature Termination of the Trial

The trial can be terminated prematurely by the coordinating investigator in the event of

- serious adverse events
- changes in the risk-benefit considerations, e.g. as a result of unexpected adverse events
- new insights from other trials
- an insufficient recruitment rate

The final decision regarding the premature termination of the trial will be made by the coordinating investigator.

4 TRIAL SUBJECTS

4.1 Inclusion Criteria

- BMI ≥27 to ≤60 kg/m^2
- T2DM
  - for patients BMI ≥35 kg/m^2: any T2DM
  - for patients BMI < 35 kg/m^2:
    - unsatisfactory medical non-insulin treatment (multiple medications, HbA1c >6.5%, hypo- or hyperglycaemias) or
    - planned insulin treatment or ongoing insulin treatment
- males and females, age ≥18 years
- written informed consent

4.2 Exclusion Criteria

- any chronic inflammatory or malignant disease
- type 1 diabetes (C-Peptid < 0,5 µg/l, GAD-Ab or ICA-Ab positive)
- peptic ulcer
- contraindication for general anaesthesia
- drug or alcohol abuse
- untreated thyroid dysfunction
- pregnancy or breast feeding woman
- fertile women (within two years of their last menstruation) without appropriate contraceptive measures (implanon, injections, oral contraceptives, intrauterine devices, partner with vasectomy) while participating in the investigation
- expected non-compliance
- participation in other interventional trial

5 TRIAL INTERVENTION

5.1 Standard RYGB (stRYGB) group and Changed limb length RYGB (cll RYGB) group

Prior to surgery all candidates will be put on a low calorie (protein rich) diet for 14 days.

Roux-en-Y gastric bypass (stRYGB and cllRYGB) reduces the size of stomach to a small pouch – about the size of a walnut (pouch volume 10-20 cm³), by stapling off a section of it. The surgeon then attaches this pouch directly to the small intestine, bypassing most of the rest of the stomach and the upper part of the small intestine.

All patients in the stRYGB group will undergo surgery with pouch size: 10 to 20 cm³ and limb length:

- BMI ≥ 50 kg/m²: alimentary limb: 170 cm, biliopancreatic limb: 80 cm).
- BMI ≤ 50 kg/m²: alimentary limb: 150 cm, biliopancreatic limb: 50 cm).

All patients in the cllRYGB group will undergo bypass surgery with pouch size: 10 to 20 cm³ and limb length:

- BMI ≥ 50 kg/m²: alimentary limb: 80 cm, biliopancreatic limb: 170 cm).
- BMI ≤ 50 kg/m²: alimentary limb: 50 cm, biliopancreatic limb: 150 cm).

RYGB (stRYGB and cllRYGB) will be done as a laparoscopic surgery. Laparoscopic surgery is performed using several small incisions, or *ports*: one to insert a surgical telescope connected to a video camera, and others to permit access of specialized operating instruments. The surgeon views his operation on a video screen.

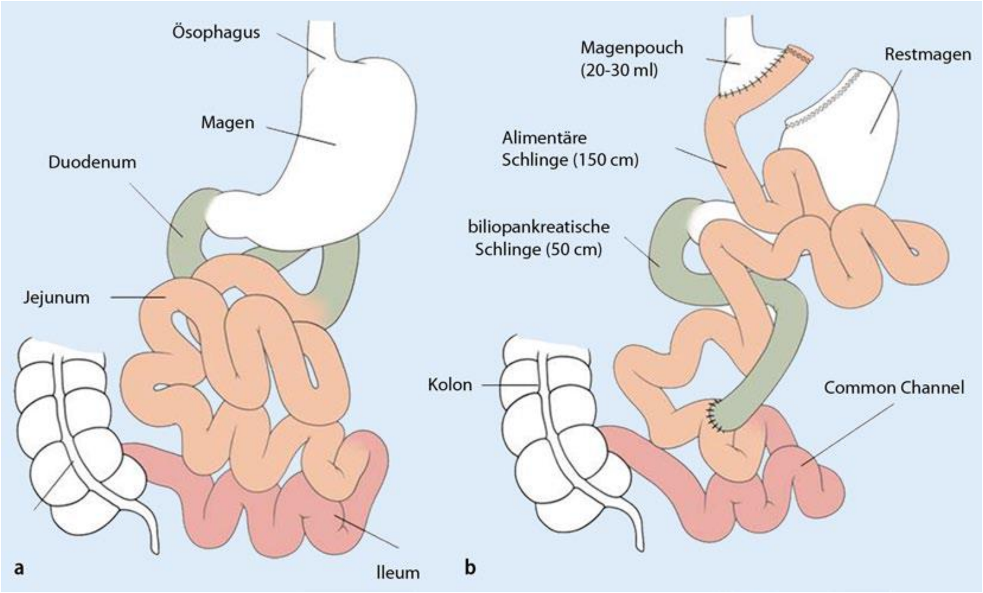

Grafic 1: Standard RYGB (stRYGB)

Due to the reduced size of the newly created stomach pouch and reduced food intake, adequate nutrition demands that the patient follow the surgeon's instructions for food consumption, including the number of meals to be taken daily, adequate protein intake, and the use of vitamin and mineral supplements. Calcium supplements, iron supplements, protein supplements, multi-vitamins (sometimes pre-natal vitamins are best), and vitamin B_{12} (cyanocobalamin) supplements are all very important to the post-operative bypass patient.

5.2 Observational Control (Medical treatment group)

Patients in this group will undergo standard medical treatment for T2DM, RYGB is possible for the patient after completing the trial at 12 months.

Irrespective of the study group, all patients will be treated with antidiabetic agents as recommended by current guidelines [8], in addition to recommendations for a suitable diet and exercise. The therapies will be prescribed by an endocrinologist specialized in diabetes treatment.

In addition to patients, willing to take part in the study, patients (only year 2016) out of the [redacted] loss program [redacted] with the [redacted] registered by ethical vote [redacted]. For these patients the duration of Visit 5 is 12 (-1; +6) month.

6 INDIVIDUAL TRIAL PROCEDURES

6.1 Screening

All patients that fulfil the inclusion/exclusion criteria and are potential candidates for surgery will be asked to participate in the trial. Those who decline to participate will be documented in the screening failure list along with the reason. To facilitate recruitment, endocrinological/diabetological practices will refer patients to the study centre. The screening failure list will be filled out at these practices.

6.2 Patient Information and Informed Consent

Patients will be asked to take part in the study in an outpatient visit. During this visit, potential candidates will be informed about the study and receive information and the consent form. Within a few weeks, patients will be asked to decide whether or not to enrol and questions will be answered prior to agreement to take part in the study. Patients who refuse surgical treatment or where health insurance rejects the application for it but want to participate in the study, can assigned in the observational control group.

The original consent forms will be stored at the trial site; one copy will remain with the patient’s clinical sheets and one copy is given to the patient.

The patient’s consent refers explicitly to the collection and processing of health-related data. The patient will be informed explicitly about the purpose of collecting the data and scope of what is to be collected and that personal data, in particular those related to health, will be used. All patients are informed about the pseudonomyzed use or evaluation of the collected data.

6.2.1 Withdrawal of Informed Consent

Patients may withdraw their consent to participate at any time without giving reasons. Nevertheless, the patient should be asked for the reason of the premature termination after being informed that he/she does not need to do so. Information as to when and why a patient was randomized and when he/she withdrew consent must be retained in the documentation.

The patient is to be informed that in case of revocation of his/her consent, the stored data may be used further, as may be necessary to

- guarantee that the patient’s personal interests are not adversely affected,
- comply with the requirement to provide complete authorisation documentation.

6.3 Enrolment in the Trial (Baseline-Visit) and Randomisation

Intervention-Groups:

Immediately after the screening (inclusion/exclusion criteria) and agreement to take part in the study, the patient will be discussed at the interdisciplinary “Board for Bariatric Surgery” and in case of a vote for the study inclusion the responsible medical insurance company will be asked to cover the treatment expenses. After a positive answer of the insurance company to cover the expenses for RYGB the baseline examinations will be performed (see flow chart) and the patient’s data will be given to the trial site.

Patients will be randomised by the trial site and the patients will immediately receive information as to which group they belong to by one of trial staff members.

Observational Control-Group:

All Patients who fulfil the inclusion/exclusion criteria, and

1. admitted to a conservative weight loss programm within the AOP IV-Vertrag in 2016, or

2. received a negative answer of the insurance company to cover the expenses for RYGB or refuse the bariatric surgery and are willing to take part in the study,

will be included in the observational control group (without randomisation).

Examinations will be performed according to the protocol of the interventional groups.

Eligibility of enrolment will be confirmed, and randomisation will be performed at visit 1 (week 0 of the study).

During the enrolment and randomisation visit, the following procedures will be performed

- Obtain medical history, comorbidities, concomitant medication and vital signs and perform physical examination (body weight, BMI, WHR, pulse, RR etc.)
- Routine blood sample (fasting glucose, fasting insulin, fasting c-peptide, HbA1c, serum lipids, Adipokines, inflammatory markers, markers od kidney and liver function etc.)
- Indirect calorimetry (performed either by a nurse or a physician, trained and experienced in this procedure)
- Pregnancy test
- Ultrasound scan (liver, fibroscan, IMT)
- MRI examination by a trained radiologist
- LiMAx test (a functional liver test, will be performed by a medical doctor before and after the praeoperatrive diet)
- BIA-measurement (performed by a trained nurse)
- Diet protocol
- Assess detailed quality of life (SF36) and physical activity (IPAQ)
- Assess Adverse Events since intervention

Whenever blood samples are taken, the scientist or another instructed (laboratory) co-worker will be present to ensure proper further sample management (storage, transport etc.). All samples will be sent to the appropriate lab and stored for further examination.

A detailed description of each procedure is provided in the Working Instructions (WIs).

Randomisation procedure (RYGB)

If a patient has signed the Informed Consent form and is eligible for the trial the investigator will proceed as follows:

- Assignment of a Patient-ID: In the patient identification list (PIL, see investigator site file), fill in the patient personal data at the first free position of the list. The Patient-ID printed in the list at this position is assigned to the patient and will be used throughout the study as a pseudonym for the patient.
- Login at the randomisation-site, preferably via the MS Internet Explorer. The user will be asked for the Patient-ID, for check of eligibility criteria, presence of informed consent in writing and stratification items. Enter these items and then click the randomisation button. The system will return to you the intervention arm of the patient.

Study-Interventions (stRYGB and cIlRYGB):

Two weeks' prior the bariatric surgery, patients undergo a low energy and protein-rich diet. The time interval between baseline/randomisation and bariatric surgery should not exceed 8 weeks. Details see chapters 5.1.

Discovery of a Violation of the Eligibility Criteria after the Fact

In general, the violation of eligibility criteria is not a reason for premature withdrawal of the patient from the trial therapy or from the whole trial.

If after randomisation it is discovered that the patient was not eligible at the time of randomisation, this has to be reported to the KKS-Data Management as soon as possible. The KKS-Data Management informs the investigator immediately as to what is to be done with the patient. The patient's data will continue to be recorded.

According to the consent form, data or tissue will be stored as long as the patient agrees.

If the patient does not meet the inclusion criteria or has to be excluded from the study, the follow up will be performed according to the study protocol as long as the patient agrees (intent to treat). Otherwise, all patients will undergo the routine bariatric follow-up as long as they show up at our clinic.

6.4 Description of further Study Visits

Visit 2 (postinterventional after 4 weeks (± 1 week))

During Visit 2, the following procedures will be performed:

- Obtain comorbidities, concomitant medication and vital signs and perform physical examination (body weight, BMI, WHR, pulse, RR etc.)
- Routine fasting blood sample (fasting glucose, fasting insulin, fasting c-peptide, HbA1c, serum lipids, Adipokines, inflammatory markers, markers od kidney and liver function etc.)
- Assess Adverse Events since intervention

Visit 3 (postinterventional after 3 months (± 1 week))

During Visit 3, the following procedures will be performed:

- Obtain comorbidities, concomitant medication and vital signs and perform physical examination (body weight, BMI, WHR, pulse, RR etc.)
- LiMAx test

- MRI examination by a trained radiologist
- Routine fasting blood sample (fasting glucose, fasting insulin, fasting c-peptide, HbA1c, serum lipids, Adipokines, inflammatory markers, markers od kidney and liver function etc.)
- Assess Adverse Events since last visit

Visit 4 (postinterventional after 6 months (± 2 weeks))

During Visit 4, the following procedures will be performed:

- Obtain comorbidities, concomitant medication and vital signs and perform physical examination (body weight, BMI, WHR, pulse, RR etc.)
- Routine fasting blood sample (fasting glucose, fasting insulin, fasting c-peptide, HbA1c, serum lipids, Adipokines, inflammatory markers, markers od kidney and liver function etc.)
- BIA-measurement (performed by a trained nurse)
- Indirect calorimetry
- Ultrasound scan (liver, fibroscan, IMT)
- MRI examination by a trained radiologist
- LiMAx test
- Assess Adverse Events since last visit

Visit 5 (postinterventional after 12 months (± 1 month))

During the Visit 5, the following procedures will be performed in all patients:

- Obtain comorbidities, concomitant medication and vital signs and perform physical examination (body weight, BMI, WHR, pulse, RR etc.)
- Routine fasting blood sample (fasting glucose, fasting insulin, fasting c-peptide, HbA1c, serum lipids, Adipokines, inflammatory markers, markers od kidney and liver function etc.)
- Indirect calorimetry
- Assess Adverse Events since last visit
- Ultrasound scan (liver, fibroscan, IMT)
- MRI examination by a trained radiologist
- BIA-measurement (performed by a trained nurse)
- Diet protocol
- Assess detailed quality of life (SF36) and physical activity (IPAQ)

6.5 Premature Termination of the Therapy or Follow-up

The date (as exactly known as possible) and if possible the circumstances and reasons for every premature termination of the therapy or follow-up will be reported to the KKS-Data Management.

Non-compliance is a common issue in trials. We define non-compliance as significant deviation from the study protocol, specifically missing more than two study visits. All patients, whether compliant or not, shall be followed-up completely.

6.5.1 Premature Termination of the Therapy for Individual Patients

The study participation will be interrupted or terminated if

- the physician decides to
- the patient cannot be reached for more than two missed visits
- if informed consent is withdrawn
- the patient becomes pregnant.

Patients excluded from the study will be reported to the KKS-Data Management. The information will include the date, when known, circumstances and reasons, whether the patient is available for further follow up investigations and whether he/she is available for the routine bariatric follow up or not.

6.5.2 Premature Termination of the Follow-up for Individual Patients

All such events will be documented in the trial sheets and at the “Prüfzentrum”.

7 METHODS OF DIAGNOSTICS AND DATA SAMPLING

Indirect calorimetry:

Resting energy expenditure will be measured by indirect calorimetry after an overnight fast. This will be performed either by a nurse or a physician, trained and experienced in this procedure. Patients will lie down and rest, once settled down, they will breathe into a plastic hood and oxygen intake and carbon dioxide output will be measured for about 20 min.

Impedance measurements (Bioimpedance Analysis, BIA):

Using this method, parameters such as body compartments total body water, fat-free mass, body cell mass, extracellular mass and fat mass will be analyzed.

Via MRI scans the development of fat areas (total and relation of subcutaneous and visceral fat) and liver fat content will be investigated. The MRI scan will consist of 48 slices between the upper diaphragm and the femur head and a liver scan. Time required: 30 min

Via Ultrasound, the liver, liver fibrosis (fibroscan) and IMT will be investigated. Time required: 20 min

Blood sample:

Whenever blood samples are taken, the scientist or another instructed (laboratory) co-worker will be present to ensure proper further sample management (storage, transport etc.). All samples will be sent to the appropriate lab and stored for further examination.

The abundance of serum proteins will be measured by ELISA or Selected Reaction Monitoring based Assays by Linear Ion-Trap Mass Spectrometry (QTRAP 5500). In addition, blood samples will be investigated for glucose, insulin, adipokines, lipids, parameters of inflammation, haematology, biochemistry etc. as mentioned above.

8 ADVERSE EVENTS (AE/SAE)

8.1 Adverse Events (AE)

8.1.1 Definitions

The Declaration of Helsinki and the ICH-GCP Guideline E6 (point 4.11 and 5.17) set the protection of participants in clinical trials in the first place. Therefore, the safety and harmlessness of interventions and therapies without drugs have to be proved in clinical trials.

Adverse Events (AE) are all in patients occurring adverse medical events, unintended disease or injuries or undesirable clinical diagnoses (including abnormal laboratory results), regardless of whether these are related to the investigation or not.

8.1.2 Documentation of adverse Events

All AE`s will be documented on the documentation sheet (CRF-page AE), including start and end of the AE, intensity, reason and final result outcome.

Patients will be asked at each visit about AE's and they are also requested to report any to the study management.

In case of Serious Adverse Events (SAE) **an** AE-sheet including additional SAE-questions will be used.

8.2 Serious Adverse Events (SAE)

8.2.1 Definitions

Serious adverse events (SAEs) are according to ICH-Guideline E2A, part IIB defined as follows:

- **results in death**,
- **is life-threatening**,

Note: The term "life-threatening" in the definition of "serious" refers to an event in which the patient was at risk of death at the time of the event; it does not refer to an event which hypothetically might have caused death had it been more severe.

- **requires in-patient hospitalization or prolongation of existing hospitalization**,
- **results in persistent or significant disability/incapacity**.

8.2.2 Documentation of Serious Adverse Events (SAE)

In case of Serious Adverse Events (SAE) an AE-sheet including additional SAE-questions will be used.

8.3 Safety Analyses

Each patient is closely monitored for safety during the study period.

8.4 Concomitant disease

Any new concomitant disease (except those listed above in 8.1.1.) or worsening of pre-existing concomitant disease will be documented as AE.

8.5 Therapeutic Procedures

If a patient requires treatment as a result of an Adverse Event, then it must meet the recognized standards of medical care in order to restore the patient's health. Appropriate resuscitation devices and medication must be available in order to treat the patient as quickly as possible in the event of an emergency.

The action taken to treat the AE/SAE must be documented by the investigator either in the appropriate CRF and/or using additional documents.

9 BIOMETRY

9.1 Randomisation

The following methods against bias will be applied:

Randomization will be stratified according to:

1. BMI
   a. ≥27 to <50 kg/m²
   b. ≥50 to ≤60 kg/m²
2. Current insulin treatment
   a. yes
   b. no
3. Sex
   a. male
   b. female

The randomization will be performed centrally by the IFB Data Center and will be computer-assisted, using Pocock's minimization algorithm (1). Before start of recruitment, simulations will be performed to demonstrate that the randomisation procedure given the stratification parameters above yields balanced results. A randomisation ratio of 1:1 will be used.

9.2 Outcomes

9.2.1 Primary Outcome

- HbA1c values after 1 year

9.2.2 Secondary Outcomes

The main secondary outcomes are:

- T2DM remission rate after 1 year <u>(only for surgical groups)</u>
  Definition T2DM remission: HbA1c <6.5% without indication for anti-diabetic medication
- Weight loss
- General well-being
- Anthropometry (body weight, BMI, WHR (waist to hip ratio), Body fat mass (BIA))
- Clinical parameters (fasting glucose, fasting insulin, fasting c-peptide, HOMA-IR index, serum lipids)

Further scientific secondary endpoints are:

Anthropometric parameter
- Ratio subcutaneous to visceral fat (MRI)

- Intima-media-thickness (IMT) A. carotis com.
- Liver volume, function (ELF-test) and morphology
- Indirect calorimetry

Blood parameters

- Serum concentration of GLP1, PYY, GIP, ghrelin (total and active), obestatin, leptin, leptin BP, adiponectin, chemerin, fetuin-A, Nampt, RBP4, Vaspin, OBBP, betatrophin (presently favored to be examined; extra serum will be frozen and stored for potentially new future parameters)
- Inflammatory markers: CrP, WBC, IL-6, TNFα

Lifestyle parameter

- Average caloric intake per day
- Change in general well-being (SF-36 questionnaire)
- Change in physical activity (IPAQ)

9.2.3 Safety Outcomes

Safety will be assessed by tabulating adverse and serious adverse events. A comparison of complications directly related to the interventions will be provided.

9.3 Statistical Description of the trial hypothesis

9.3.1 Primary Outcome

HbA1c

H_0: The mean value of HbA1c after 1 year is independent of the treatment arm

H_A: The mean value of HbA1c after 1 year depends on the treatment arm

9.3.2 Secondary Outcomes

T2DM remission

H_0: The proportion of patients with diabetes remission after one year is independent of the treatment arm.

H_A: The proportion of patients with diabetes remission after one year depends on the treatment arm.

Weight loss

H_0: The course of weight over time, measured as a percentage of the baseline value, does not depend on the treatment arm.

H_0: The course of weight over time, measured as a percentage of the baseline value, depends on the treatment arm.

Treatment complications

Estimates for the proportion of procedure related complications will be provided for the treatment arms separately along with confidence intervals.

General well-being

H_0: The mean SF-36 score after 1 year is independent of the treatment arm

H_A: The mean SF-36 score after 1 year depends on the treatment arm

9.4 Sample Size Discussion

On the basis of the important trial by Schauer et al. 2012, the HbA1c-levels can be expected to decrease in the pooled surgery groups by 2.9 (1.7) points and in the conservative group by 1.4 (1.5) points. This already takes into account the expectation of lower initial values for HbA1c in our trial, which, however, has almost no effect on the sample size calculation – only the relative difference between arms matters.

Choosing a 5% significance level, 17 conservative and 119 surgical patients have to be analysed using a t-test to reach 95% power for demonstrating this difference. The analysis with ANCOVA will have higher power still. Expecting a drop-out-rate of 15%, 24 + 68 + 68 patients have to be included, to be analysed: 20 + 60 + 60 (control + stRYGB + clIRYGB).

Even with a pessimistic scenario in which the changes in the surgical group are 1.4 points vs 0.4 points with conservative therapy, the ANCOVA analysis still yields a power of 89%.

With the above sample size and pooling the variance between surgical arms, the width for the confidence interval when comparing the two surgical arms can be expected to be smaller than 1.2 percentage points.

9.5 Statistical Methods

9.5.1 Analysis Population

The main analyses will be performed for the per-protocol (PP) population in all three groups, including the observational arm. Only patients who received the planned treatment and provide data for the primary endpoint will be counted amongst the PP population.

Secondary analyses will be performed for the intention to treat (ITT) population in all three groups, including the observational arm. Missing HbA1c data will be accounted for using multiple imputation. If a patient does not provided data regarding diabetes remission, then this patient will be treated as not having had remission in the analysis.

9.5.2 Planned Methods for Analysis

Efficacy: Comparison of the interventions with respect to the HbA1c levels after 1 year

Description of the primary efficacy analysis and population: The primary endpoint will be analysed using ANCOVA for the HbA1c levels, with the baseline value as a covariate and the treatment arm and stratification categories as factors. A closed testing procedure will be used for multiple comparisons. Thus, the three comparisons between the arms will be performed at the nominal significance level of 5% if the global test result is significant. Because the comparison of the surgical groups is of such interest, this comparison will be performed regardless of the result of the global test, though a Bonferroni correction will be used if the global test is not significant.

Secondary endpoint: The comparison of the diabetes remission rates between the surgical arms will make use of a chi-squared-test, and an estimate with 95% confidence interval for the relative probability (“risk”) of remission rates will be provided.

9.6 Interim Analysis

No interim analysis will be performed.

9.7 Final Analysis

Final analysis will be performed when all patients finished the final study visit, all queries were

answered and the study database was closed.

10 ETHICAL, LEGAL AND ADMINISTRATIVE ASPECTS

10.1 GCP-Statement

All persons participating in the conduct of the trial commit themselves to observe the Declaration of Helsinki in the current version, as well as all pertinent national laws and regulations, the ICH guidelines for Good Clinical Practice (GCP) issued in June 1996.

10.2 Submission

The trial can start only after obtaining a positive review by the ethics committee. The written approval of the EC must be filed in the trial master file (TMF). Additionally, every participating centre must receive a copy of these documents to be filed in the investigator site file (ISF).

10.3 Protocol Amendments

Changes made to the protocol that was appraised positively by the ethics committee must be positively reappraised if the changes

- are such that they may affect the subjects' safety, e.g.
- result in further data collection that necessitates changes to the patient information and/or informed consent form,
- affect the interpretation of the scientific documents upon which the trial is based or the significance of the results of the trial,
- significantly affect the leadership or conduct of the trial.

Changes in the trial protocol can only be made by a committee, including the Coordinating investigator, data management and biometry.

11 DOCUMENTATION

11.1 Case Report Forms (CRF)

The clinical CRF will be provided by the [redacted] electronic form. Investigators will connect to the database via internet and input data directly into the eDE database. However, a paper version of the CRF will be available, so data can be documented in case of malfunction of the electronic system. The content of this paper version will then be entered to the database later. Each CRF page should be completed as soon as possible after the respective visit. Each CRF page will be signed electronically. The signatures serve to attest that the information contained in the CRF is true and has not been falsified.

The questionnaires of quality of life, physical activity and the diet protocol will be provided to the patient in paper form and these date will be entered into the eCRF by trial personnel afterwards.

At all times the local principal investigator has final responsibility for the accuracy and authenticity of all clinical and laboratory data entered in the CRF.

As source data are regarded:

- for clinical or demographic data: the patient file
- for resting energy expenditure, MRT and ultrasound data: the print-out
- for questionnaire: the questionnaires / diary filled out by the patients
- laboratory print outs for fasting glucose, fasting insulin, HbA1c and Serum lipids (total cholesterol, HDL-cholesterol, LDL-cholesterol, free fatty acids, triglycerides)

All other scientific data will be transferred directly into the electronic data base.

11.2 Data Management

For creation of the study database the EDC Tool eData Entry (eDE) by OmniCom will be used. The database will be validated according to the Standard Operating Procedures (SOPs) of the [redacted] prior to data capture.

The information entered into the database by the investigators or an authorised member of the study team is systematically checked for completeness, consistency and plausibility by routines implemented in eDE, running every night. Error messages generated by these routines will by checked by the data management staff. Queries obviously not representing a true problem will be closed. Errors with an obvious solution will be corrected by the KKS staff immediately (self-evident correction).

Discrepancies, errors or omissions will be passed to the investigator or an authorised member of the study team at the investigational site by the query management tool of eDE. The investigator will receive notification of all queries concerning his/her investigational site. The [redacted] will supervise and support the solution of the queries. Corrected data will be re-checked by automatic routines during the night after entry. In case a query cannot be solved, the Data Management staff of th[redacted] may close the query. This shall happen in agreement with the study biometrician and clarification, if the information addressed by the query is relevant for the results of the study, or not.

During the whole course of the study, a backup of all data is made on a daily basis. Unauthorized access to patient data is prevented by the access concept of the study database which is based on a strict hierarchy and role model. Any change of data (e.g. when data is changed in the database during query management) is recorded automatically via audit trail within the database.

At the end of the study, once the database has been declared complete and accurate, the database will be locked. Thereafter, any changes to the database are possible only by joint written agreement between coordinating investigator, biometrician and data manager.

In 2022 all data will be transferred to the [redacted] Conditions as above will be continued.

11.3 Archiving

All relevant trial documentation (Trial Master File), the electronically stored data, the original CRFs and the final report will be stored for at least 10 years [redacted]s completion.

At the investigating sites, the investigators' files, patient identification lists, signed written consent forms, copies of all CRFs and the patients' files will be stored for at least 10 years after the trial's completion.

12 SUPERVISION OF THE CLINICAL TRIAL

12.1 Access to Source Data

According to ICH-GCP and the applicable German laws, the principal investigator must permit all authorized third parties access to the trial site and the medical records of the trial subjects (source data). These include the clinical trial monitors, auditors and other authorized employees of the sponsor, as well as members of the local or federal authorities. All these persons are sworn to secrecy.

12.2 Monitoring

To monitor the study center, on-site monitoring by staff of [redacted] is planned.

Following the appropriate analyses according to the principles of risk analysis a risk-adapted monitoring according to class K2 will be performed with respect to the monitoring strategy (frequency/degree of SDV) after.

During the initiation visit at the trial site an introduction to the course of studies, ISF, logistics, SAE management and electronic data entry is done.

Regular monitoring visits are organized according to the quality of the trial site up to three times a year. Scope of these visits is to check compliance of the trial site with the study protocol and GCP rules. For all patients, the informed consent documents will be checked. In addition, source data verification (SDV) of the key data (eligibility criteria, intervention, outcome measures) will be performed routinely in a random sample of 50% of the patients. Details are described in the Monitoring Manual. Monitoring will follow the [redacted] SOPs.

At the end of the study, a final visit is carried out.

A Trial Steering Committee consisting of principal and promoting investigator, biometrician, project management and physician will be established, in order to monitor and to supervise the progress of the trial, and to ensure adherence to the protocol. A meeting of the committee will be scheduled every 6 months.

The interventions (stRYGB and clIRYGB) used in this clinical trial are minimally invasive clinically proven therapies for the treatment of type 2 diabetes. The application of these therapies performed by experienced physicians in clinical practice. Therefore, a Data Monitoring Safety Board is not deemed necessary in this trial.

12.3 Independent Supervision of the Trial

We will prepare standard operating procedures for all methodologies involved in treatment and diagnostic methods of this proposed trial. The study will be conducted according to Good Clinical Practice Guidelines (www.ich.org) and all study investigators will sign an according statement.

The trial will be performed in cooperation with the IFB Data Centre, which is located at Clinical Trial Centre [redacted]

13 DATA PROTECTION AND CONFIDENTIALITY

All legal requirements concerning data protection and confidentiality will be respected.

Within this study, personal data from the trial subjects and data regarding the therapy and the course of disease (medical results, types of therapy, medication etc.) will be collected.

The data will be stored and processed in pseudonymized form (i.e. without reference to the patient's name) with the aid of an identification number.

Data will be analysed at the [redacted] The safety concept ensures amongst other things that data access is limited to authorized persons, that measures are taken to prevent loss of data and that the laws pertaining to data protection are observed. The data are protected from third party access and only members of the trial are permitted access. These members are sworn to secrecy.

In the event of withdrawal of consent, the necessity for storing data will be evaluated. Data not needed will be deleted immediately. Personal data will be stored in an anonymous manner after reaching the study aim 10 years at the latest, if there are no other regulatory or contractual time periods for archiving.

Declaration regarding Data Protection

During data entry, processing and analysis in the [redacted] all requirements of the data protection act will be taken into account. Access to the data is strictly limited to authorized persons. Data are protected against unauthorized access.

14 ADMINISTRATIVE AGREEMENTS

14.1 Adherence to the Protocol

The clinical trial described here will be conducted and analysed in accordance with ICH guidelines for Good Clinical Practice (GCP).

Protocol violations are all deviations from the procedures outlined in this document, such as

- Study visits that are missed or that take place at the wrong time
- non-compliance and so on.

After a patient has been enrolled, it is the investigator's responsibility to avoid protocol violation in order to obtain unbiased data for the trial.

Major protocol violations will be reported to the coordinating investigator immediately. All protocol violations will be documented and discussed with the responsible biometrician before closing the data bank and carrying out the statistical analysis.

The investigator must ensure that the recorded data are documented as per protocol. Minor variations are inevitability, but must be documented together with a justification.

14.2 Funding and Insurance

Study funding:

[redacted]

Specifics regarding the study insurance can be found in the Trial Master File.

14.3 Publication Policy and Registration

A major goal of this study is to find relevant scientific and clinical data. These data have to be published as soon as possible, in a journal ranked as highly as possible.

Authorship and co-authorship will be given to everybody who made significant contributions to the paper. Others will be listed in the Acknowledgement.

The authors are obliged to call the [redacted] in the byline. In the institutional allocation, the wording [redacted] to use.

[redacted]

In citable abstracts, it should be analogous to the conveyor and the IFB obesity. In addition, on posters, the logo of the IFB Adiposity Diseases and the BMBF must be used.

The data generated in this study are generally open to others within the IFB for further use.

The study is registerd [redacted]

15 CONCOMITANT SCIENTIFIC PROJECT

15.1 LiMAX-Test

The LiMAx-Test will be added to the trial investigations

One of the organs we focus on is the liver, a central metabolic organ. Obesity and Typ 2 diabetes are the major risk factors for nonalcoholic fatty liver disease (NAFLD), which may lead to nonalcoholic steatohepatitis (NASH), fibrosis and finally cirrhosis.

The LiMAx test is the best currently available test, reflecting the liver function.

Study intention:

1. Identification of patients with increased risk for surgery (liver function limitations)
2. Investigation of the effect of the preoperative hypocaloric protein rich diet and the bariatric procedure on the liver function.
3. Will obese T2DM patients (with NASH driven impaired liver function) benefit from above preoperative diet in general prior major surgery?
4. Do morphological parameters (Ultrasound, Fibroscan, MRT) reflect the expected functional changes?

The LIMAX test will be performed per patient four times: before and after the preoperative diet, and 3 and 6 months after surgery (Visits 3 and 4). The test will always be performed by or under the supervision of a medical doctor.

10 patients out of each operative group (total of 20 patients) and 10 patients out of the control will be asked to undergo the LiMAx test. They will be matched as well as possible regarding BMI, sex, age and diabetes stage to have the option to compare the two operative arms.

Description of the LiMAx test: Non-radioactive labelled methacetin solution will be injected i.v.. In healthy liver cells, methacetin is metabolized into labelled carbon dioxide and a small amount of the painkiller paracetamol. The resulting labelled carbon dioxide is specifically detected by the FLIP medical device in the exhaled air. Based on the amount of the exhaled

carbon dioxide the FLIP device calculates a value – the LiMAx value – which depends on liver function. Therefor it is necessary to breathe about 15 to 30 minutes in a face mask, connected to the FLIP medical device.

The LiMAx-Test is already established routine test in our clinic to evaluate the liver function prior to liver resections. Patients suffering obesity and type 2 diabetes are at an increased risk of suffering impaired liver function due to NASH, fibrosis or cirrhosis.

For the LiMAx test, patients will receive a separate sheet for patient information and agreement.

The evaluation of the LiMAx test as above is supported by the Humedics GmbH (Humedics GmbH, Marie-Elisabeth-Lüders-Straße 1, 10625 Berlin), covering the test expenses. This is an additional test to above MetaSurg study. The IFB administration agreed and there are no problems with the additional financial support.

15.2 MRI (brain and spinal cord) and cognitive function

When performing the abdominal MRI, an MRI of the brain and spinal cord and tests for cognitive function will be added to the trial investigations.

That part of the study will be run in cooperation of the [redacted] Max Planck Institute for Human Cognitive and Brain Sciences.

Recent findings suggest that brain structure and function correlate with obesity and food intake. Also, a certain loss of grey and white matter volume and alterations in microstructure are described (when compared to normal BMI subjects) in the literature. However, little or nothing is known about the effects of obesity/metabolic surgery in this context and the mechanisms behind that.

We plan to investigate up to 20 patients. It is not possible, to estimate the effect size so far due to a lack of controlled trials. However, as bariatric surgery induces a massive change in body weight and metabolism, the estimated related change in brain structure and function seems likely rather large, and therefore detectable with this sample size using sensitive high-resolution neuroimaging.

Study intentions:

1. Does (regional) brain inflammation correlate with systemic inflammation and / or visceral fat mass and will brain inflammation decrease after obesity surgery?
2. Will (impaired) cognitive function improve after obesity surgery?
3. Will (impaired) total (and grey matter) brain / spinal cord mass increase after obesity surgery?
4. Mechanisms of 1 to 3? Do brain or cognitive function changes correlate with systemic inflammation, visceral fat mass, microbiota or any serum parameter / hormones etc.

When undergoing the abdominal MRI, additional T1- and diffusion-weighted brain and spinal cord sequences will be run. Altogether, the examination will take 15 to 20 more minutes.

All new patients (randomized surgical and observational control) will be asked to undergo the additional sequences und to complete a paper-and pencil (TMT) and a computer-assisted task (Mnemonic Similarity Task [a Test for Assessing Hippocampal Integrity]).

The Max Planck Institute will pay a patient fee of €200 per patient as reimbursement, after undergoing all brain MRI´s and fully completing the questionnaires at all times points.

16 PROTOCOL SIGNATURES

Confirmation of the Final Protocol

We hereby certify that this is the final version of the protocol:

Principle Investigator:	______________________	______________________
	Date	Signature
Biometrician:	______________________	______________________
	Date	Signature

17 PROTOCOL AGREEMENT

Herewith I declare that I have read and understood the present protocol and agree to honour each part of it. I will ensure that all the patients enrolled in the trial by my site will be treated, observed and documented in accordance with this protocol. I will ensure that all persons assisting with the study under my supervision are adequately informed about the protocol, the investigational product and their duties.

Date: ______________________

Signature of Principal Investigator: ______________________

Affiliation/address (stamp): ______________________

____

____

____

18 APPENDIX

18.1 Classification of Adverse Events

18.1.1 Degree of Severity

The degree of severity of an Adverse Event will be determined in accordance with the definitions in 7.1.

18.1.2 Assessment of Intensity

The assessment of the intensity accords with CTCAE V4.0

Mild Adverse Event	• asymptomatic or mild symptoms;<br>• clinical or diagnostic observations only;<br>• intervention not indicated.
Moderate Adverse Event	• minimal, local or noninvasive intervention indicated;<br>• Only non-invasive measures is required
Severe Adverse Event	• medically significant but not immediately life-threatening;<br>• hospitalization or prolongation of hospitalization indicated;
Life-threatening Adverse Event	• Life-threatening consequences;<br>• exacerbated by an acute, life-threatening complications of metabolism or the circulatory system, e.g. circulatory collapse, haemorrhage, sepsis<br>Need for intensive care, immediate invasive, interventional radiology, or measures, therapeutic endoscopy or surgery
Death related to Adverse Event	

18.1.3 Determining the Causal Relationship

The investigator must assess whether or not the Adverse Event is causally related to the administration of the trial intervention. The following classification is to be used.

- Reasonable possibility
- No reasonable possibility

A reasonable possibility exists, if one of the following WHO-UMC criteria is met:

- occurring in a plausible time relationship, and which cannot be explained by concurrent disease or other drugs or chemicals
- with a reasonable time sequence, unlikely to be attributed to concurrent disease or other drugs or chemicals
- with a reasonable time sequence, but which could also be explained by concurrent disease or other drugs or chemicals
- more data is essential for a proper assessment
- cannot be judged because information is insufficient or contradictory

No reasonable possibility exists, if the following WHO-UMC criterion is met:

- with a temporal relationship which makes a causal relationship improbable, and in which other drugs, chemicals or underlying disease provide plausible explanations.

18.1.4 Expected/Unexpected

Adverse Events are unexpected if they do not occur in the manner or with the intensity described in the SmPC/Investigator's Brochure (see investigator's files).

18.1.5 Outcome of an Adverse Event

The outcome of an Adverse Event is classified as follows:

- recovered/resolved
- recovering/resolving
- not recovered/not resolved
- recovered/resolved with sequelae
- fatal*
- unknown

*Note: A patient's death is not in itself an event, but the consequence of one. The event that led to the patient's death must be documented completely and reported even if death occurs four weeks after stopping study intervention and independent of whether or not there is a relation to the therapy or not.

18.2 Acronyms

Ab	Antibody
AE	adverse event
ANCOVA	analysis of covariance
BIA	Bioimpedance Analysis
BMI	body mass index
CRF	case report file
ELF-test	extended liver function test
EC	Ethic committee
FFM	fat free body mass
GCP	Good Clinical Practice
GIP	gastric inhibitory peptide
ICH	International Conference on Harmonization
IFB	Integriertes Forschungs- und Behandlungszentrum
IMT	intima-media-thickness
ITT	intention to treat
KKS	Koordinierungszentrum für Klinische Studien
MRI	Magnet resonance imaging
REE	resting energy expenditure
PP	per protocol
RYGB	Roux-Y-Gastric Bypass
SAE	serious adverse event
SAR	serious adverse reaction
SDV	Source Data Verification
SOP	standard operating procedure
T2DM	Type 2 diabetes mellitus
TMF	trial master file
WHO-UMC	World Health Organization – Uppsala Monitoring Centre
WHR	waist to hip ratio
WI	working instruction

[redacted]

19 REFERENCES

1. Buchwald H, Avidor Y, Braunwald E et al, Bariatric surgery: a systematic review and meta-analysis. JAMA. 2004. 13;292(14):1724-37.
2. G Schauer PR, Sangeeta R, Kashyap MD at al., Bariatric Surgery versus Intensive Medical Therapy in Obese Patients with Diabetes. N Engl J Med 2012; 366:1567-1576
3. S3-Leitlinie: Chirurgie der Adipositas. http://www.adipositas-gesellschaft.de/fileadmin/PDF/Leitlinien/ADIP-6-2010.pdf
4. Cohen R, le Roux CW, Papamargaritis D et al, Short Report: Pathophysiology Role of proximal gut exclusion from food on glucose homeostasis in patients with Type 2 diabetes. DIABETICMedicine, DOI: 10.1111/dme.12268, 2013
5. Rodriguez L, Reyes E, Fagalde P, et al. Pilot clinical study of an endoscopic, removable duodenal-jejunal bypass liner for the treatment of type 2 diabetes. Diabetes Technol Ther 2009;11:725–32.
6. Schouten R, Rijs CS, Bouvy ND, et al. A multicenter, randomized efficacy study of the EndoBarrier Gastrointestinal Liner for presurgical weight loss prior to bariatric surgery. Ann Surg 2010;251:236–43.
7. De Jonge C, Rensen SS, Verdam FJ, et al. Endoscopic duodenal-jejunal bypass liner rapidly improves type 2 diabetes. Obes Surg 2013;23:1354–60.
8. Nationale VersorgungsLeitlinie Therapie des Typ-2-Diabetes. http://www.deutsche-diabetes-gesellschaft.de/fileadmin/Redakteur/Leitlinien/Evidenzbasierte_Leitlinien/NVL-DM2-Ther-lang_Endversion_270813.pdf
9. Tarnoff M, Rodriguez L, Escalona A, et al. Open label, prospective, randomized controlled trial of an endoscopic duodenal-jejunal bypass sleeve versus low calorie diet for pre-operative weight loss in bariatric surgery. Surg Endosc 2009; 23:650–6.
10. Escalona A, Pimentel F, SharpA et al. Weight Loss and Metabolic Improvement in Morbidly Obese Subjects Implanted for 1 Year With an Endoscopic Duodenal-Jejunal Bypass Liner. Ann Surg 2012; 255:1080–1085
11. Shimizu H, Timratana P, Schauer PR, Rogula T. Review of Metabolic Surgery for Type 2 Diabetes in Patients with a BMI < 35 kg/m2, J Obes 2012; Article ID: 147256, doi: 10.1155/2012/147256
